# DNA methylation changes and increased mRNA expression of coagulation proteins, factor V and thrombomodulin in Fuchs endothelial corneal dystrophy

**DOI:** 10.1007/s00018-023-04714-x

**Published:** 2023-02-11

**Authors:** Ida Maria Westin, Mattias Landfors, Antonios Giannopoulos, Andreas Viberg, Pia Osterman, Berit Byström, Sofie Degerman, Irina Golovleva

**Affiliations:** 1 Department of Medical Biosciences, Medical and Clinical Genetics, Umeå University, Umeå, Sweden; 2 Department of Medical Biosciences, Pathology, Umeå University, Umeå, Sweden; 3 Department of Integrative Medical Biology, Umeå University, Umeå, Sweden; 4 Department of Clinical Sciences, Ophthalmology, Umeå University, Umeå, Sweden; 5 Department of Clinical Microbiology, Umeå University, Umeå, Sweden

**Keywords:** Fuchs dystrophy, Transcription factor 4 (*TCF4*), DNA methylation, Trinucleotide repeat disorder, Coagulation factors, Factor V, Thrombomodulin

## Abstract

Late-onset Fuchs endothelial corneal dystrophy (FECD) is a disease affecting the corneal endothelium (CE), associated with a cytosine-thymine-guanine repeat expansion at the CTG18.1 locus in the *transcription factor 4* (*TCF4*) gene. It is unknown whether CTG18.1 expansions affect global methylation including *TCF4* gene in CE or whether global CE methylation changes at advanced age. Using genome-wide DNA methylation array, we investigated methylation in CE from FECD patients with CTG18.1 expansions and studied the methylation in healthy CE at different ages. The most revealing DNA methylation findings were analyzed by gene expression and protein analysis. 3488 CpGs had significantly altered methylation pattern in FECD though no substantial changes were found in *TCF4*. The most hypermethylated site was in a predicted promoter of aquaporin 1 (*AQP1*) gene, and the most hypomethylated site was in a predicted promoter of coagulation factor V (*F5* for gene, FV for protein). In FECD, *AQP1* mRNA expression was variable, while *F5* gene expression showed a ~ 23-fold increase. FV protein was present in both healthy and affected CE. Further gene expression analysis of coagulation factors interacting with FV revealed a ~ 34-fold increase of thrombomodulin (*THBD*). THBD protein was detected only in CE from FECD patients. Additionally, we observed an age-dependent hypomethylation in elderly healthy CE.Thus, tissue-specific genome-wide and gene-specific methylation changes associated with altered gene expression were discovered in FECD. *TCF4* pathological methylation in FECD because of CTG18.1 expansion was ruled out.

**Supplementary Information:**

The online version contains supplementary material available at 10.1007/s00018-023-04714-x.

## Introduction

The corneal endothelium (CE) is a monolayer of cells on the posterior surface of the cornea that has a crucial role in maintaining the cornea in a semi-dry optical transparent state. Fuchs endothelial corneal dystrophy (FECD) is a bilateral disease caused by progressive loss of corneal endothelial cells, which at an advanced stage leads to corneal swelling and vision loss [1]. Hallmark of FECD is thickening of the Descemet’s membrane due to excessive accumulation of extracellular matrix and the presence of guttata; best described as penetrating droplets of extracellular matrix in the CE from the posterior collagenous layer of Descemet’s membrane [2, 3].

Two forms of FECD are known so far, one manifesting at a young age and therefore denoted early-onset FECD, and the second form starting later in life, termed late-onset FECD. Early-onset FECD is associated with mutations in *COL8A2* and the disease is often advanced in the fourth decade of life [4–6]. Late-onset FECD with corneal guttata observed from the fourth decade in life and onward, was initially associated with an intronic SNP; rs613872 in *transcription factor 4* gene (*TCF4*) [7]. Later, an even stronger association was found to a cytosine-thymine-guanine (CTG)_n_ repeat expansion in an intron of *TCF4*, known as the CTG18.1 locus and located 43 kb from the original SNP [8]. Today, multiple studies on the *TCF4* repeat expansion and FECD have been conducted in several populations, all showing convincing association to FECD with repeat lengths over 40–50 [9–17]. Expansion of the CTG18.1 locus in the *TCF4* gene makes FECD one of the most common tri-nucleotide repeat disorders along with myotonic dystrophy (DM1 and DM2), Huntington disease (HD), Spinocerebellar ataxia (SCA), Friedreich ataxia (FA) and Fragile X syndrome (FRAXA) [18].

*TCF4* is a member of the class I basic-helix-loop-helix (bHLH) family of transcription factors, that bind to the DNA motif called E-box (‘CANNTG’) and modulate transcription. These E-box regulatory sites found in promoters and enhancer elements of numerous genes regulate tissue-specific gene expression [19].

One of the proposed pathogenic mechanisms in FECD is the development of tissue-specific RNA nuclear foci formed from the expanded *TCF4* repeat tract. The RNA foci present in CE cells were shown to sequester splicing factors such as Muscleblind Like Splicing Regulator 1 (MBNL1) and Muscleblind Like Splicing Regulator 2 (MBNL2) [20–22]. Another proposed mechanism is the repression of certain micro-RNAs that normally suppress the expression of extracellular matrix components in the posterior layer of the cornea, leading to excess of extracellular matrix [23–25].

In other trinucleotide repeat disorders, such as DM1, FA and FRAXA, an increase in DNA methylation upstream of the repeat expansion tracts was suggested as a pathogenic mechanism. These diseases are characterized by large expansions (> 100 repeats) in non-coding sequences, and their hypermethylation affects gene expression [26–31]. The CTG18.1 locus in *TCF4* is also located in non-coding sequence within intron 2 (NM_001083962.2), and the median *TCF4* repeat length is close to 100 repeats in the Swedish FECD cohort [17]. A well-known consequence of DNA methylation at gene promoter regions is gene silencing, whereas gene body methylation may be involved in the regulation of gene splicing [32, 33] and affect gene expression through i.a. enhancers [34, 35]. DNA methylation patterns in CE tissue from FECD patients with unknown *TCF4* status have been investigated earlier, revealing altered methylation of promoter regions in FECD patients compared to controls [25, 36]. However, the effect of an expanded CTG18.1 locus on methylation in FECD remains unknown. Moreover, global hypomethylation is thought to contribute to age-related diseases such as degenerative joint diseases, cancer and progressive neurodegenerations [37–39], but the methylation pattern in the aged CE has never been investigated.

In this study, we performed a genome-wide DNA methylome analysis as well as targeted gene expression analysis in FECD patients and controls to gain deepened knowledge in FECD biology. Also, we specifically investigated whether an expanded CTG18.1 locus affects the *TCF4* methylation in CE.

## Materials and methods

### Patients and controls

In this study approved by the Swedish Ethical Review Authority (2019–01,744), we used specimens consisting of corneal endothelial layer (CE) with Descemet’s membrane and white blood cells (WBCs) drawn from peripheral blood from FECD patients. All FECD CEs were taken during routine corneal transplantation using Descemet Stripping Automated Endothelial Keratoplasty (DSAEK) method [40, 41] and all patients had undergone cataract surgery with phacoemulsification and posterior chamber intraocular lens prior to transplantation. The patients received both oral and written information about the study before transplantation and written informed consent was obtained. CEs from non-FECD corneal donors were used as controls. These healthy human corneas were from deceased individuals who had chosen, when alive, to donate their corneas postmortem, through written consent and according to Swedish law. Three of the donors had undergone cataract surgery when alive. The donated corneas were kept in the Eye Bank at the Tissue Establishment in the University Hospital of Umeå, Sweden. If these healthy donated corneas were not used for transplantation after their collection, they were delivered to the laboratory for research purposes. All human specimens were handled under the guidelines based on the tenets of the Declaration of Helsinki developed by the World Medical Association (2013).

### Biological samples

During the DSAEK of included FECD patients, the central 8 mm CE and Descemet’s membrane were removed with a Reversed Sinskey Hook after Trypan blue staining (descemetorhexis) and placed in RNAlater (Invitrogen, Waltham, USA).

**Fig. 1:**
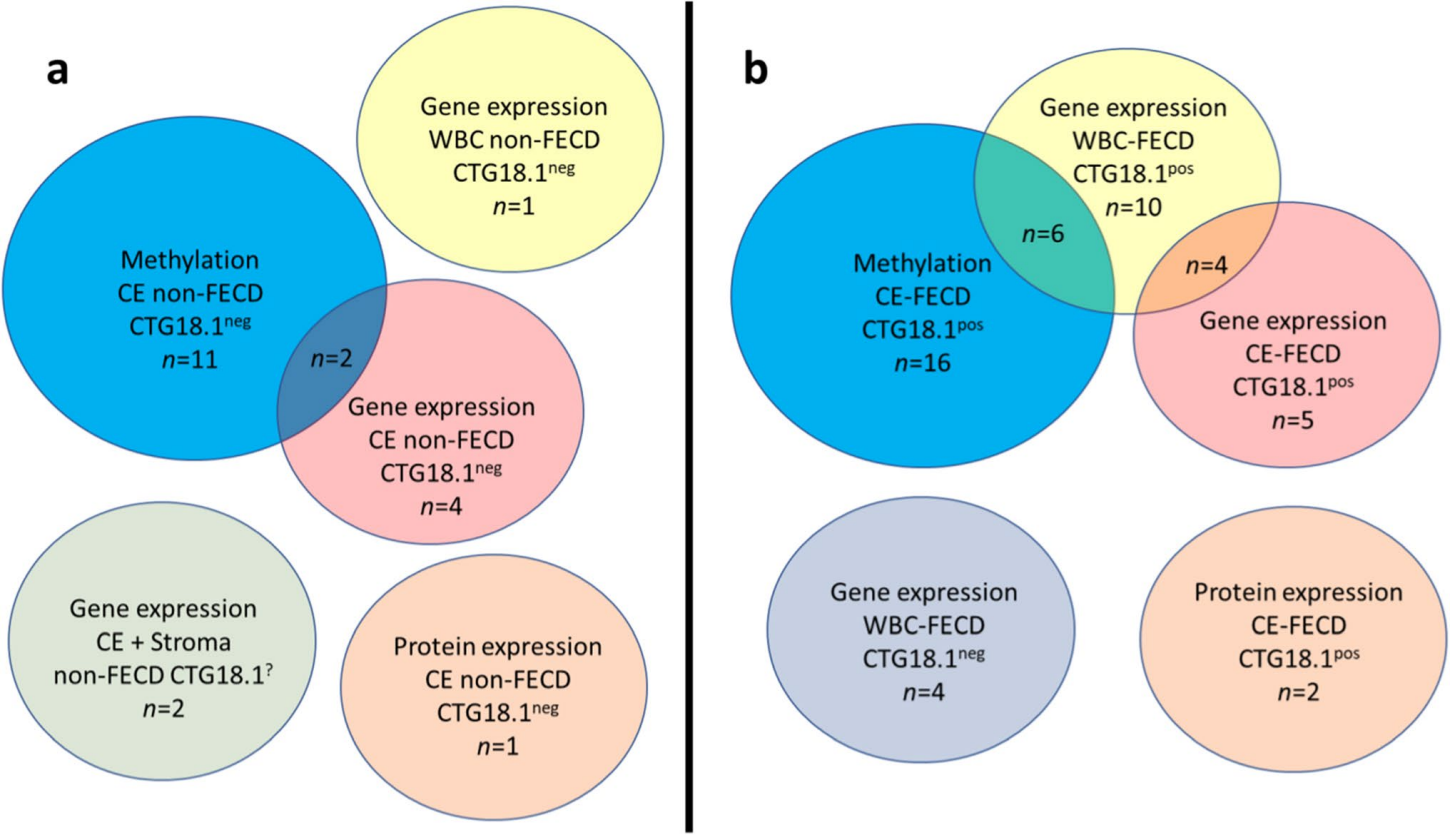
Schematic overview of the number of individuals used in the different assays with (**a**) display of non-FECD controls and (**b**) display of FECD patients. Overlapping circles represent numbers of paired samples between assays. CE—corneal endothelium, WBC—white blood cells, CTG18.1^pos^—individuals harboring *TCF4* CTG18.1 > 40 repeats, CTG18.1^neg^—individuals harboring *TCF4* CTG18.1 < 40 repeats, CTG18.1^?^ —individuals with unknown *TCF4* CTG18.1 repeat length

The corneal tissues from non-FECD donors (controls) were stored in the Eye Bank in a nutritional solution for an average of 62.8 (± 34.7) days before DNA extraction. The nutritional solution was mixed in 500 ml volume, consisting of GIBCO’s Minimum Essential Medium (MEM) (Thermo Fisher Scientific, Waltham, USA) with 8% GIBCO’s Fetal Bovine Serum (Thermo Fisher Scientific), 100 mg Biklin (Vianex S.A., Athens, Greece) 1.25 mg Fungizone, (Cheplapharm, Greifswald, Germany), 12.5 mM GIBCO’s HEPES (Thermo Fisher Scientific) and 1 g/0.125 g Piperacillin/Tazobactam (Fresenius Kabi AB, Bad Homburg, Germany). The corneal endothelial layer together with its basement membrane was detached from the cornea and placed in RNAlater (Invitrogen, Waltham, USA). The remaining corneal tissue from non-FECD donors was placed into a separate RNAlater vial and used for *TCF4* CTG18.1 genotyping. All corneas were washed twice with phosphate-buffered saline (PBS) and frozen at -80 °C on the same day of surgery or upon receiving corneal donor tissue. DNA extracted from CE of 16 FECD patients and eleven non-FECD donors (controls) were used for methylation array analysis (Fig. 1, Online Resource 1, Table S1). RNA extracted from 19 specimens from FECD patients, and seven samples from non-FECD controls were used for gene expression analyses. Both WBC, CE and stroma were used in the gene expression assays, and some samples were paired with the CE used in the methylation analysis (Fig. 1).

### DNA extractions

Genomic DNA from human CE was extracted using NucleoSpin Tissue XS (Macherey–Nagel, Düren, Germany). The DNA was eluted in 20 µl BE buffer. Because of limited access to CE and the low yield of DNA extracted from CE cells, DNA concentration was only measured in one sample by spectrophotometry with DeNovix DS-11 FX (DeNovix, Wilmington, USA) to get an estimate of the extraction method yield. Genomic DNA from WBC was extracted using a modified standard salting-out-method [42, 43].

### Methylation array analysis

The EZ DNA Methylation kit (Zymo Research, Irvine, USA) was applied for bisulfite conversion of genomic DNA from CE tissue according to the protocol, with the recommended temperature profile for Illumina Infinium Methylation assay: 16 cycles of 95 °C for 30 s and 50 °C for 60 min with a final step at 4 °C. The bisulfite-converted DNA was eluted in 12 µl M-elution buffer.

To confirm successful bisulfite conversion, a MethyLight analysis of the *ALU* gene with ALU-C4 primer probe set was used [44]. Modifications were made to the MethyLight qPCR reaction: 1X TaqMan Universal MasterMix II (no UNG) (Applied Biosystems, Waltham, USA), 0.6 µM ALU-C4 forward primer, 0.6 µM ALU-C4 reverse primer, 0.2 µM ALU probe (6-FAM, NFQ-MGB) and 1 µl of bisulfite converted DNA. The qPCR program was set at 40 cycles instead of 50 on QuantStudio 6 real-time PCR system (Applied Biosystems). DNA extracted from cell line CCRF-CEM (CCL-119™, American Type Culture Collection, Manassas, Virginia, USA) was also bisulfite converted and used as a standard curve in five tenfold serial dilutions ranging from 50 ng to 0.005 ng.

Genome-wide DNA methylation data were obtained by using Human Infinium MethylationEPIC array (Illumina, San Diego, USA). Briefly, 4 µl of speed-vac concentrated bisulfite-converted human CE DNA was used for array analysis according to the manufacturer’s protocol. The array was scanned with a HiScan array reader (Illumina), and the methylation level of each CpG site was determined by calculating the ratio (i.e., the *β* value) between the intensity of the methylated alleles and the total intensity using the minfi package version 1.32 [45]. The *β* value ranges from 0 (completely unmethylated) to 1 (fully methylated). Normalization was done with BMIQ version 1.3 [46], and annotation and pre-filtering were done in R version 3.6.2. The pre-filtering steps excluded CpGs with detection P-value > 0.05, CpG probes that aligned to multiple loci in the genome or were located less than 5 bp from a known single nucleotide polymorphism [47], and CpGs probes identified as meQTLs [48, 49]. CpGs with missing values in any of the samples were removed, and X and Y chromosomes were omitted from the analysis to avoid sex-related methylation biases. The genome build used was GRCh37/hg19. The previous dataset (GEO GSE94462) from [36] was also analyzed separately according to our method.

### Genotyping and sanger sequencing

The assay to determine the *TCF4* CTG18.1 repeat lengths for all samples included in this study (except two samples of CE with corneal stroma) was performed as described earlier [17]. Throughout this study, expanded *TCF4* CTG18.1 alleles were defined as consisting of more than 40 (CTG)_n_ repeats.

In addition, the FECD patients analyzed by methylation assay were genotyped with TaqMan® SNP Genotyping Assay (Thermo Fisher Scientific) for two common polymorphisms in coagulation factors. Commercially available assay C_11975250_10 (Thermo Fisher Scientific) was used for targeting rs6025 (chr1:169519049TG > A) known as factor V Leiden, (*F5* for gene, FV for protein) and a custom design was used for targeting rs1799963 (chr11:46761055G > A) in coagulation factor 2/prothrombin), *F2* (F: 5’- GTGTTTCTAAAACTATGGTTCCCAATAAAAGT-3’, R: 5’- CCATGAATAGCACTGGGAGCATT -3’, Probe VIC mutant: 5’- TCTCAGCGAGCCTC -3’, Probe FAM wildtype: 5’- CTCAGCAAGCCTC -3’).

Sanger sequencing was performed in all FECD patients and non-FECD controls analyzed with methylation array; 79 bps upstream and 150 bps downstream of *F5* probe cg13122356 (F: 5’-GGGCAAAGGTCTGATTCACA-3’, R: 5’-GGGGCAGGACAAGTTAGAAC-3’) and 215 bps upstream and 199 bps downstream of *AQP1* probe cg03310518 (F: 5’-CTCTCTTTCATGACCTGGGG-3’, R: 5’-TCCTACTTCTCAGAGCCCAG). In short, PCR amplified fragments were cleaned with Exonuclease I (Thermo Fisher Scientific) and sequencing reactions were set up with BigDye™ Terminator v3.1 Cycle Sequencing Kit (Applied Biosystems) according to the manufacturer’s protocol. Sequencing products were run on the ABI3500 Dx genetic analyzer (Applied Biosystems) and data were evaluated using Sequencher software version 4.9 (Gene Codes Corporation, Ann Arbor, USA).

### RNA extractions

Peripheral blood was collected in whole blood vacutainer tubes with EDTA. WBCs were lysed in a buffer containing 130 mM NH_3_Cl, 2 mM NH_3_HCO_3_ and 0.02% diethylpyrocarbonate (DEPC). Total RNA from WBC was extracted using TRIzol Reagent™ (Invitrogen). For extraction of total RNA from the CE from FECD patients and non-FECD controls, miRNeasy Micro Kit (Qiagen, Hilden, Germany) was used.

### cDNA synthesis and gene expression

The reverse transcriptase reaction was done according to the manufacturer’s instructions with SuperScript™ IV VILO™ Master Mix with ezDNase™ Enzyme (Invitrogen). For each tissue type and each RNA extraction method, a “no reverse transcriptase control” (no RT) was included to verify the absence of genomic DNA contamination in subsequent analysis.

FAM- labeled TaqMan^®^ gene expression assays (Thermo Fisher Scientific) were used to quantify coagulation factor V, *F5* mRNA (Hs00914120_m1, targeting NM_000130.4), aquaporin, *AQP1* mRNA (Hs01028916_m1, targeting NM_198098.2, NM_001185060.1, NM_001185061.1, NM_001185062.1 and NM_001329872.2), coagulation factor II, prothrombin, *F2* mRNA (Hs01011988_m1, targeting NM_000506.4 and NM_001311257.1), coagulation factor II thrombin receptor, *F2R* mRNA (Hs00169258_m1, targeting NM_001992.4), coagulation factor III, tissue factor, *F3* mRNA (Hs01076029_m1 targeting NM_001178096.1 and NM_001993.4), coagulation factor X, *F10* mRNA (Hs00984442_m1 targeting NM_000504.3, NM_001312674.1 and NM_001312675.1), protein C, coagulation factor XIV, *PROC* mRNA (Hs00165584_m1 targeting NM_000312.3) and fibrinogen alpha chain, *FGA* mRNA (Hs00241027_m1, targeting NM_000508.4 and NM_021871.3) at exon–exon boundaries. Thrombomodulin, *THBD* mRNA was also assayed (Hs00264920_s1 targeting NM_000361.2); however, it consists of only one exon and therefore exon–exon boundary detection is unachievable.

VIC-labeled probe targeting exon–exon boundaries in RAS-associated protein, *RAB7A* mRNA (Hs01115139_m1, targeting NM_004637.5) was chosen as an internal reference gene due to its stable expression in 52 human tissues and cell lines [50].

Droplet digital PCR (ddPCR) reactions were run in 20 µl reactions with 1 × ddPCR Supermix for Probes (no dUTP) (Bio-Rad, Hercules, USA), 1 × TaqMan probe (FAM), 1 × TaqMan probe (VIC) and 4 ng cDNA. For droplet generation, Droplet Generation Oil for probes (Bio-Rad) and QX200 Droplet Generator (Bio-Rad) were used. The PCR program was set as follows; initial denature step at 95 °C for 10 min, 40 cycles of; 94 °C for 30 s and 60 °C for 1 min (ramp rate -2 °C/sec), ending with 98 °C for 10 min and 4^0^C infinity. QX200 Droplet Reader (Bio-Rad) was used for droplet detection and the Absolute Quantification (ABS) was used as the detection method. QuantaSoft software version 1.7.4.0917 was used for analysis. For all assays, absolute values of copies/µl were normalized against *RAB7A*, and the expression of the gene of interest (GoI) was presented as % of ratio *GoI/RAB7A*. The “no reverse transcriptase control” was run for each tissue type to rule out genomic DNA contamination. All biological samples were run in single technical replicates. To rule out false positives, more than one biological replicate had to have at least one positive droplet or a single positive sample had to have more than one positive droplet.

### Fluorescent labeling and imaging of CE tissue

The endothelial layer attached to Descemet's membrane from one donor and two FECD patients was put in a nutrition medium described under the biological samples section.

The tissues were thereafter washed twice with PBS and fixed with 10% formalin neutral buffer for 60 min. Two washing steps with PBS were followed, and the tissues were permeabilized for 30 min at room temperature (RT) with penetration buffer (PBS, 0.2% Triton X-100, 0.3 M Glycine, 20% DMSO). Subsequently, the samples were washed for 40 min (4 × 10 min) with washing buffer (PBS, 0.2% Tween 20, 10 ug/ml heparin) and blocked for 60 min with blocking buffer (PBS, 0.2% Triton X-100, 6% Donkey serum, 10% DMSO). The recipes for penetration buffer, washing buffer and blocking buffer are adopted from Visikol (Visikol, Hampton, USA). Last, the tissues were divided into smaller pieces and were stained overnight with the primary antibodies: Thrombomodulin (1:100, MA5-11,454, Invitrogen) and factor V (1:100, PA5-81,998, Invitrogen). The next day the tissues were washed for 40 min (4 × 10 min) and incubated with secondary antibodies: Alexa fluor 488 (1:400, ab150113, Abcam, Cambridge, United Kingdom) and Alexa Fluor 594 (1:300, A32740, Invitrogen) for three hours at RT, then washed for 30 min (3 × 10 min), incubated 13 min with DAPI (1:3000, Thermo Fisher Scientific), and washed one more time for 10 min. Prior to imaging, the tissues were placed on microscope slides and a few drops of Prolong Gold Antifade Mountant (Invitrogen) were added onto the tissues before placing the coverslips, and the mounted samples were left overnight. Z-stack fluorescent images were acquired using a 40x, 1.4 numerical aperture oil-immersed lens on a Leica SP8 confocal microscope (Leica Microsystems, Wetzlar, Germany). The Z-stacks of the images were combined in a single picture with open sourced Image J software version 2.3.0/1.53f51 [51], with Java version 1.8.0_172 (64 bit), using the maximum intensity projection method.

### Statistical analysis and data visualization

Two-sided independent T-test, two-sided Mann–Whitney U and false discovery rate (FDR) with Benjamini-Hochberg [52] were calculated with Python-based open-source software Scipy version 1.5.4 (scipy.org) and Numpy version 1.16.4 (numpy.org) with Python version 3.7.2 in Jupyter Notebook version 6.0.0 with Pandas version 0.24.2 to detect significant differences between FECD patients and non-FECD controls. A CpG site was determined as differently methylated (DM-CpG) if the absolute value of the difference in beta value (Δβ) between the mean of FECD patients and the mean of non-FECD controls was ≥ 0.2 or ≤ -0.2 and if FDR q-value was < 0.05. Pearson’s Chi-squared test (*df* = 6) was calculated in R version 4.2.1 to compare the distribution of the number of CpG sites in the different target genomic regions (TSS1500, TSS200, 5’UTR, 1^st^ Exon, Gene body, 3’UTR and intergenic) on the Human Infinium MethEPIC850K array in relation to the entire Human Infinium MethEPIC850K array, and to compare it to the distribution of CpGs located in each target genomic region on the array in relation to the DM-CpGs found in FECD.

Individual Fisher's exact tests (region vs non-region) and Bonferroni adjusted P-value were calculated in R version 4.2.1 for each genomic region of the DM-CpGs (TSS1500, TSS200, 5’UTR, 1^st^ Exon, Gene body, 3’UTR and intergenic) and presented as odds ratio (OR, > 1 overrepresented, < 1 underrepresented).

Matplotlib version 3.1.0 (matplotlib.org) was used for plotting gene methylation levels and gene expression levels. Bioinfokit version 1.0.2 was used for producing volcano plots [53]. The open-sourced software CpGtools beta_PCA.py version 1.0.9 was used for principal component analysis [54]. Two-sided Mann–Whitney U was used to calculate the significance in gene expression results.

## Results

### TCF4 genotyping in FECD patients and non-FECD controls

All FECD patients and non-FECD controls included in this study were tested for CTG18.1 expansion in the *TCF4,* except for two anonymous non-FECD donor’s corneas used for gene expression analysis in the stroma. In the FECD group, successfully interrogated by methylation array 12 out of total 16 patients were females (75%) and the median age in this group was 72.5 years (IQR 16.5) (Online Resource 1, Table S1). All FECD patients had a *TCF4* CTG18.1 expansion with more than 40 repeats and the median repeat length of the expanded allele was 94 repeats (IQR 15.5). In the non-FECD control group analyzed in the methylation assay (*n* = 11), all had less than 40 *TCF4* (CTG)_n_ repeats, and the median CTG18.1 repeat length was 16 (IQR 8.0) for the longest allele (Online Resource 1, Table S1). 55% of the non-FECD methylation controls were females and the median age of the elderly non-FECD methylation controls (≥ 57 years, *n* = 9) was 78 years (IQR 17.5) while the median age of the younger controls (< 30 years, *n* = 2) was 27 years (IQR 1), and for the whole group the median was 76 years (IQR 27) (Online Resource 1, Table S1).

### Differential methylation analysis in FECD with TCF4 CTG18.1 expansion vs non-FECD controls

Initially, DNA from CE from 17 FECD patients with *TCF4* CTG18.1 expansion (*n* > 40 repeats) and from 11 non-FECD controls (donors) without *TCF4* CTG18.1 expansion (*n* < 40 repeats) were available for analysis with Human Infinium MethylationEPIC array, containing probes for ~ 850 000 CpG sites. However, one of the FECD patients (FECD case 15) was excluded from the downstream analysis due to the poor quality of the results on the methylation array, thus yielding methylation results from 16 FECD patients.

We observed an age-associated hypomethylation among the elderly non-FECD controls (≥ 57 years) compared to the younger non-FECD controls (< 30 years) (Online Resource 1, Fig. S1 and Fig. S2). Therefore, in our further analysis we used only age matched controls, and the two younger non-FECD controls were excluded from the comparison.

**Fig. 2:**
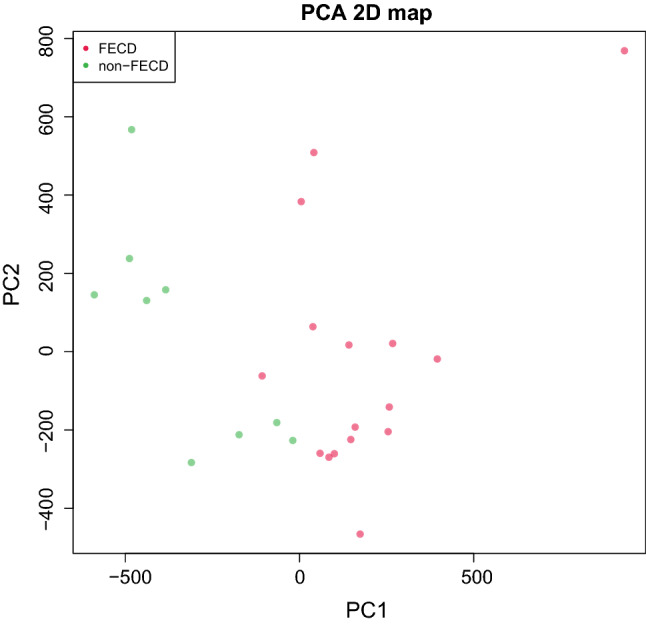
Principal component analysis of normalized methylation β-values comprising 686 247 CpG sites in corneal endothelium of FECD patients (red) and non-FECD donors (green) with projection of samples onto the first two principal components (PC) of the methylation β-value measurements

**Fig. 3:**
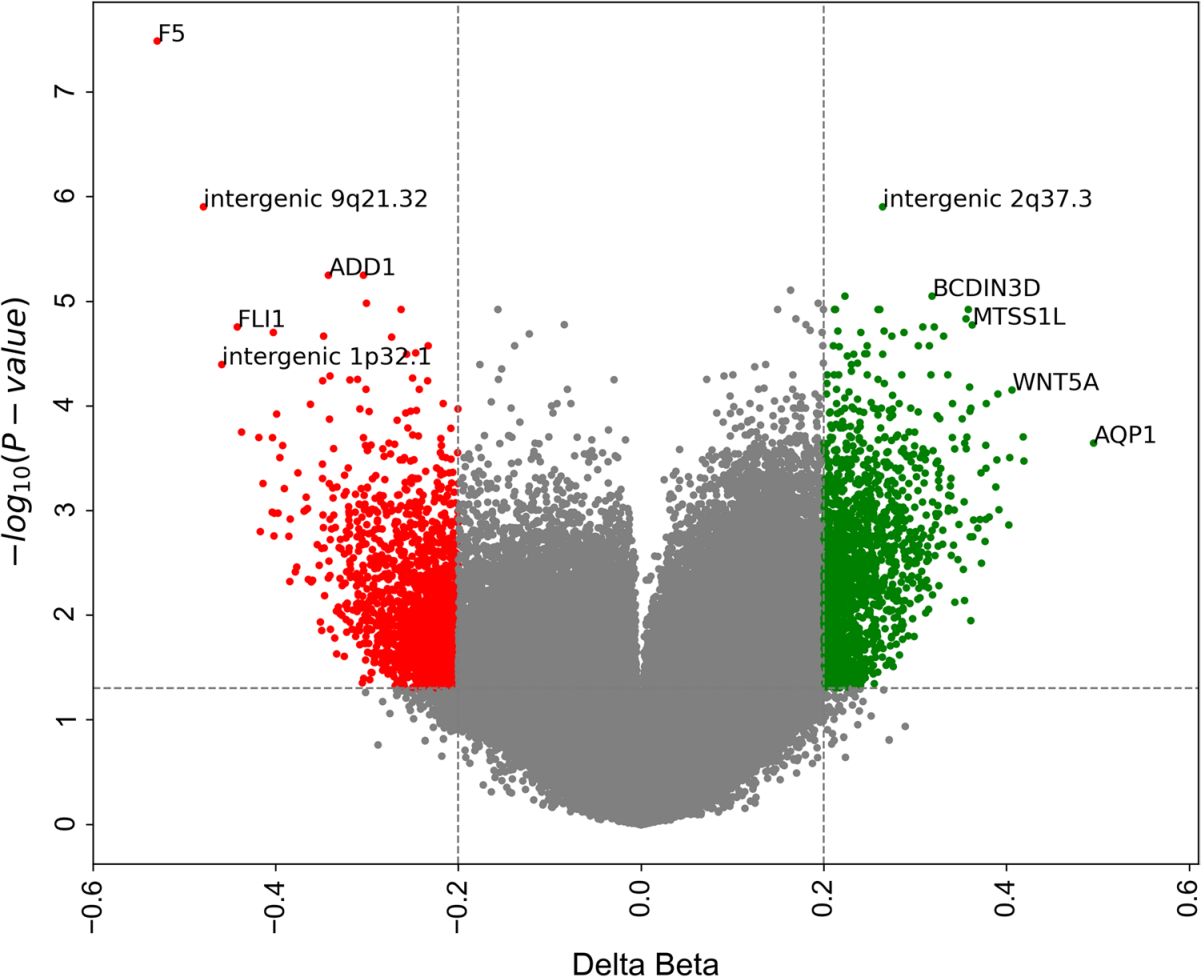
Volcano plot showing statistical significance and delta beta (Δβ) values of individual probes in the corneal endothelium (CE) from FECD patients (*TCF4* CTG18.1 > 40 repeats) compared to CE from non-FECD controls *TCF4* CTG18.1 < 40 repeats). Selected CpG extremes are displayed with gene names or genomic association. The red dots indicate CpG sites with Δβ values below -0.20 and green dots indicate CpG sites with Δβ values above 0.20 in FECD patients. The horizontal dotted line shows threshold significance of adjusted P-value 0.05, and the vertical dotted lines display thresholds of Δβ ± 0.2

**Fig. 4:**
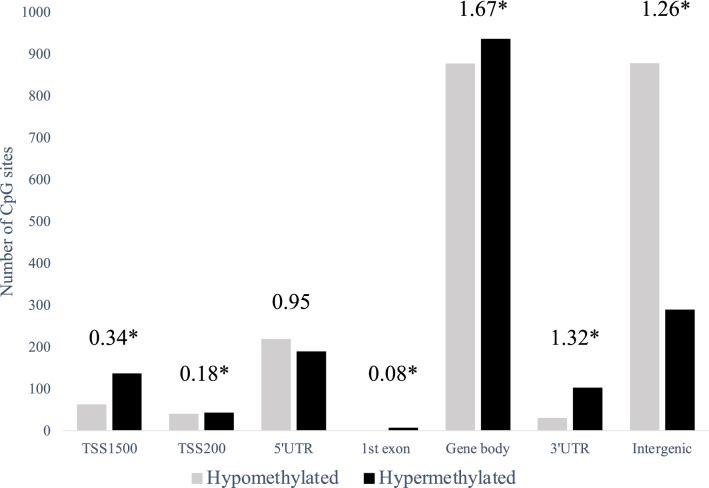
Number of significant (FDR *q* < 0.05) differentially methylated (DM) CpG sites (Δβ ± 0.2) between FECD patients and age matched non-FECD controls, grouped by targeted genomic region. Grey stacks display hypomethylated DM-CpG sites and black stacks displays hypermethylated DM-CpG sites. Numbers on top of bars indicate odds ratio. *—*P*-value < 0.001. TSS1500 – 1500 bp from transcription start site, TSS200—200 bp from transcription start site

After filtering, subset of 686 247 CpG sites remained and was used for further analysis. A principal component analysis of FECD cases (*n* = 16) and non-FECD donors (*n* = 9) revealed two subtle clusters, one for FECD cases and one for non-FECD donors (Fig. 2). The mean β-value for each CpG site was calculated, and significant differences in methylation pattern between the FECD (*n* = 16) and non-FECD control group (*n* = 9) (Δβ =  ± 0.2 with *q*-value < 0.05) were identified. In total, 3488 CpG sites were denoted as differentially methylated CpG sites (DM-CpGs) in the FECD group (*P*-value =  < 2.2e-16, Chi-square), of which 1983 CpGs were hypomethylated and 1505 CpGs were hypermethylated (Fig. 3 and Online Resource 2) (NCBI GEO accession number GSE198917). The DM-CpG sites in FECD were overrepresented in gene bodies (OR 1.67, *P*-value =  < 0.001), intergenic regions (OR 1.26, *P*-value =  < 0.001) and 3’UTR regions, (OR 1.32, *P*-value =  < 0.001) (Fig. 4), and underrepresented in TSS1500, TSS200 and 1st Exon, with OR of 0.34, 0.18 and 0.08, respectively (*P*-value =  < 0.001). No difference in the distribution of DM-CpGs in 5’UTR was found (OR 0.95) (Fig. 4). No difference in methylation with the threshold of Δβ ± 0.2 was detected in the *TCF4* gene (Online Resource 1, Fig. S3) nor 200 kb upstream or downstream of the gene.

**Fig. 5:**
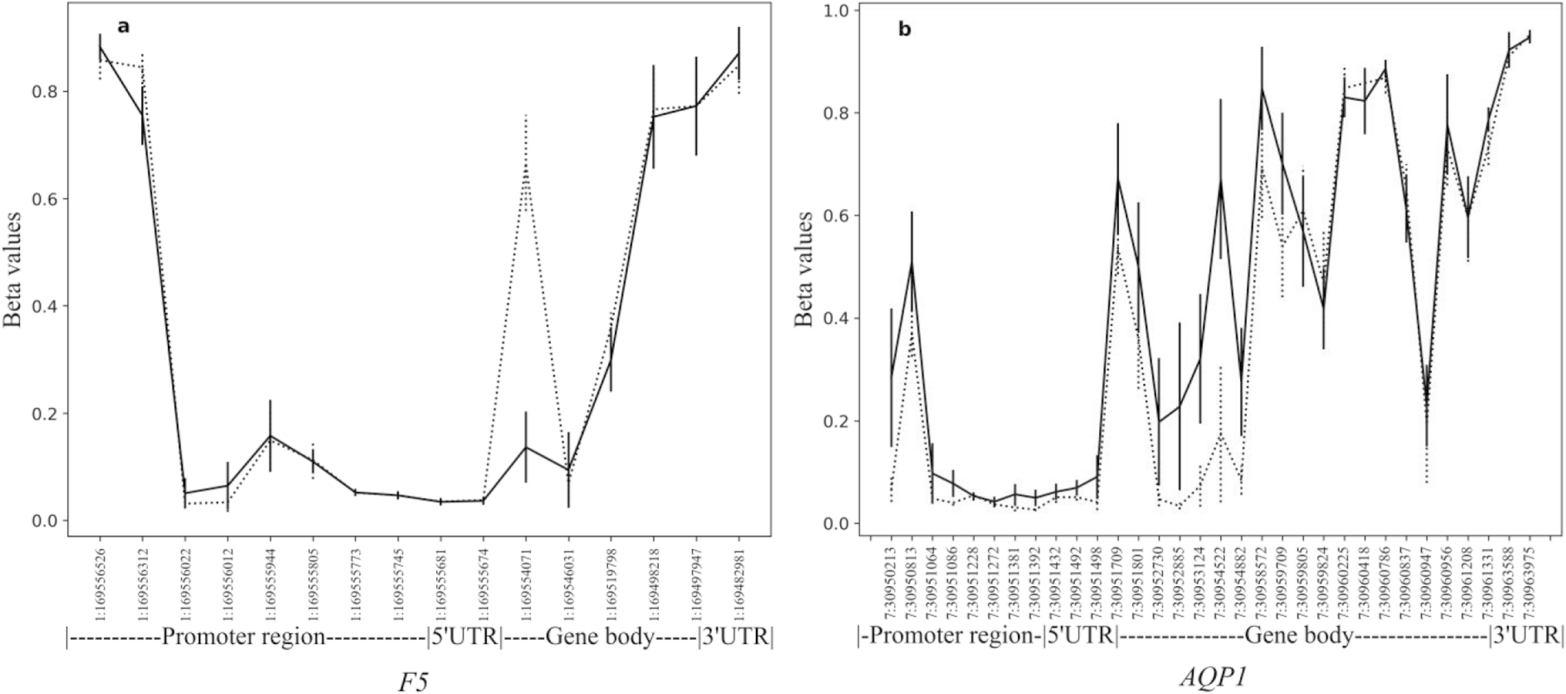
Mean methylation level (β) in the corneal endothelium from non-FECD controls (dotted line) and FECD patients (continuous line) in the (**a**) *F5* gene and (**b**) *AQP1* gene. Gene region annotation according to the MANE select and canonical transcripts NM_000130.5 (*F5*) and NM_198098.4 (*AQP1*). Vertical lines show standard deviation at each CpG site. Genomic positions are according to genome build GRCh37/hg19

The CpG site (cg13122356) with the most pronounced decrease in methylation (Δβ = − 0.53) was in the coagulation factor V (*F*5) gene, (*q*-value = 0.00817) (Fig. 5a) on chromosome 1, at position g.169554071 within the predicted promoter/enhancer GH01J169584 (genecards.org) /ENSR00000376822 (ensembl.org). In contrast, the CpG site with the maximum increase in methylation (Δβ =  + 0.495) matched the probe cg03310518 targeting the aquaporin gene, *AQP1* (*q*-value = 0.00817) (Fig. 5b). This CpG site is located on chromosome 7, at position g.30954522 within the predicted promoter/enhancer GH07J030910 (genecards.org) /ENSR00000820824 (ensembl.org).

Since all of our FECD cases had undergone cataract surgery prior to DSAEK, we wanted to rule out the DM-CpGs in *F5* and *AQP1* as a consequence of cataract surgery. Therefore, we compared elderly cataract operated non-FECD controls (*n* = 3) against elderly non-cataract non-FECD controls (*n* = 6) and found that Δβ was 0.03 for *F5* and 0.04 for *AQP1*, implying that cataract surgery does not impact *F5* and *AQP1* methylation. Furthermore, we compared cataract operated non-FECD controls (*n* = 3) against the cataract operated FECD patients (*n* = 16) and here Δβ was -0.51 for *F5* and + 0.47 for *AQP1*. These data conclude that cataract surgery is not the cause of the DM-CpGs in *F5* and *AQP1*.

To rule out SNV at or in close proximity to DM probe positions cg13122356 (*F5*) and cg03310518 (*AQP1*), all FECD patients and non-FECD controls interrogated with methylation assay were analyzed by Sanger sequencing. No common SNV that could explain the differences in methylation between controls and FECD patients was found.

Forty-three probes targeting 30 different miRNA genes demonstrated a significant level of Δβ value ± 0.2. Nine of these miRNAs, miR-33B, miRNA-3681HG, miR-548A2, miR-548F1, miR-548H4, miR-661, miR-6787, miR-7853 and miR-942 had ≥ 2 CpG sites reaching above the threshold (Online Resource 3, pp 1–5).

Genes known to be associated with FECD (*COL8A2*, ZEB1, *SLC4A11*, *AGBL1*, *LAMC1*, *LOXHD1*, *KANK4*, *ATP1B1* and *DMPK*) [5, 55–60] were analyzed for methylation differences between FECD patients and non-FECD controls. None of the CpG sites in these genes reached the significant threshold of Δβ ± 0.20 (Online Resource 3, pp 6–10).

We analyzed the GEO dataset GSE94462 [36] by running the raw data through our pipeline, and we found that probe cg03310518 in *AQP1* had a Δβ of + 0.35 in the FECD group. The probe cg13122356 in *F5* does not exist on the Illumina 450 K methylation array and could therefore not be analyzed. Also, in our dataset, we analyzed the methylation level in genes for which probes in our filtered data overlap with the top 20 probes found by Khuc et al*.* [36] (*EML3* and *MYADML*) (Online Resource 3, p11) and Pan et al. [25] (*miR199B*, *miR33B*, *miR1286*, *miR1306*, *miR130A*, *miR942*, *miR499*, *miR184*, *miR194-2*, *miR25*, *miR1471*, *miR199A1* and *miR320D1*) (Online Resource 3, pp 12–18), both studies based on the GSE94462 dataset. The probe in *EML3* had a Δβ of + 0.22 and the probe in *MYADML* had Δβ of -0.05 in our dataset (Online Resource 3, p 11). From the top 20 probes of Pan et al. study, only the probe in *miR33B* reached our threshold of Δβ of ± 0.20 at + 0.25 (Online Resource 3, pp12-18). However, for *miR942* in our dataset, two consecutive probes, downstream of the probe listed as top 20 [25], reached our significant threshold and were DM-CpGs in our study (Online Resource 3, p13).

### Gene expression of F5 and AQP1

**Fig. 6:**
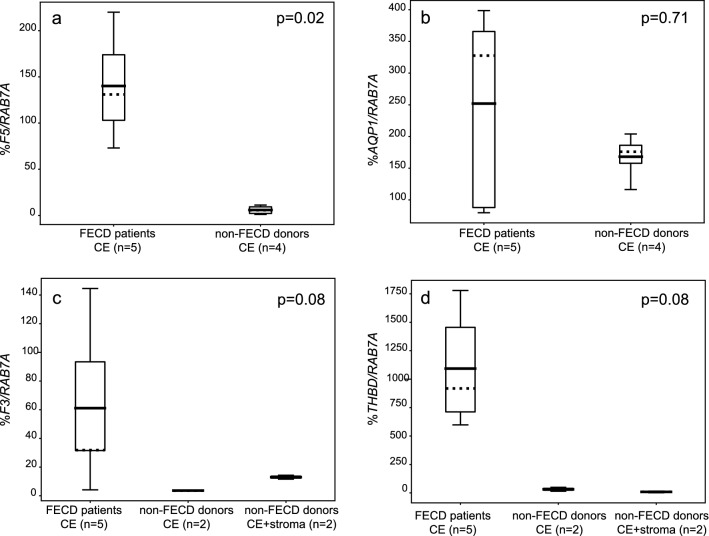
mRNA gene expression levels normalized against gene coding for RAS-associated protein (*RAB7A*) in the corneal endothelium (CE) from FECD patients and non-FECD controls for (**a**) factor V (*F5*) gene and (**b**) aquaporin 1 (*AQP1*) gene. mRNA gene expression levels normalized against *RAB7A* in CE from FECD patients, non-FECD controls and corneal grafts containing corneal stroma and CE from non-FECD controls for (**c**) tissue factor (*F3*) gene and (**d**) thrombomodulin (*THBD*) gene. *P*-values displayed are a comparison between FECD patients and CE from non-FECD controls only (not stroma). Filled horizontal lines within boxes display means, and dotted lines within boxes display medians

Considering the significant difference in methylation in the predicted promoter/enhancer regions of *F5* and *AQP1* genes in this study, we examined their mRNA expression*.* Absolute quantification of targeted genes was performed by ddPCR on RNA extracted from CE from FECD patients (*n* = 5) and from non-FECD controls (*n* = 4) (Fig. 6, Table 1). In addition, gene expression was analyzed using RNA extracted from WBC from ten FECD patients with *TCF4* CTG18.1 expansion, four FECD patients without *TCF4* CTG18.1 expansion and one non-FECD control (Table 1). Thus, FECD diagnosis and *TCF4* CTG18.1 genotype were taken into account when gene expression was analyzed in CE and WBC.

**Table 1:** mRNA gene expression of *AQP1* and coagulation factors

Specimen	*CTG18.1*^c^	*n*	*AQP1*	*F5*	*F2*	*F2r*	*F3*	*F10*	*PROC*	*THBD*	*FGA*
FECD CE	pos	5	252 ± 139	140 ± 52	0.14 ± 0.19	2.0 ± 0.9	61 ± 51	0	0	1092 ± 453	0
non-FECD CE^a^	neg	4	168 ± 32	5.9 ± 4.2	0.05 ± 0.07	1.5 ± 0.9	3.6 ± 0.1	0	0	32 ± 17	0
non-FECD CE + Stroma^b^	?	2	–	–	0	–	13 ± 1.4	1.3 ± 0.4	0	8.8 ± 7.1	0
FECD WBC	pos	10	0.14	15	0.006	3.5	0.05	0	0.01	5.8	0
FECD WBC	neg	4	0.04	11	0.003	2.9	0.14	0	0.008	6.5	0
Control WBC	neg	1	0.08	8.5	0	4.5	0.03	0	0.007	9.1	0

Gene expression was measured in different tissues and normalized against internal Ras-Associated Protein (*RAB7A*) gene expression. CE—corneal endothelium, WBC—white blood cells. See also Online Resource 4

^a^due to tissue limitation, only two donors were analyzed for *F3*, *F10*, *THBD* and *PROC*

^b^(–) indicates that not all assays were analyzed in stroma tissue

^c^(pos) indicates *TCF4* (CTG)_n_ repeat length more than 40 repeats; (neg) indicates *TCF4* (CTG)_n_ repeat length less than 40 repeats; (?) indicates unknown *TCF4* (CTG)_n_ repeat length

In our experiments, the mRNA expression of *F5* gene was ~ 23-fold higher in CE from FECD patients compared to non-FECD controls (*P*-value = 0.02) (Fig. 6a, Table 1). Moreover, *F5* expression in WBC from FECD patients was substantially lower than in FECD CE (Table 1). No significant differences were observed for *F5* expression in WBC from FECD patients with and without *TCF4* CTG18.1 expansion (Table 1). These data indicate tissue-specific increase of *F5* expression in CE from FECD patients.

The differentially methylated *AQP1* gene (hypermethylated in CE tissue from FECD patients) has previously been associated with disorders involving imbalance in ocular fluid movement [61], which is also a characteristic feature in FECD. We observed a noteworthy variation although not statistically significant (*P*-value = 0.71) in *AQP1* expression levels in CE from FECD patients compared to CE from non-FECD controls (Fig. 6b, Table 1). *AQP1* was barely expressed in WBC from FECD patients with and without *TCF4* CTG18.1 expansion and in the non-FECD control without *TCF4* repeat expansion (Table 1).

### Gene expression of clotting factors and F5, F2 genotyping

Considering that coagulation factor V (FV) is a central player in the coagulation process, involved in numerous interactions with other clotting factors, we investigated mRNA expression of its direct and indirect interacting targets. Expression of the genes coding for coagulation factor II, thrombin (*F2*), coagulation factor II, thrombin receptor (*F2R*), coagulation factor III, tissue factor (*F3*), coagulation factor X (*F10*), coagulation factor XIV, protein C (*PROC*), thrombomodulin (*THBD*) and fibrinogen alpha chain (*FGA)* was analyzed in the same tissues as for *F5* (Table 1). Barely detectable or no expression of *F2* was found in CE and WBC from FECD patients and non-FECD controls (Table 1, Online Resource 4)*.* No difference in *F2R* gene expression was detected between CE from FECD patients and CE from non-FECD controls (Table 1). *F2R* gene expression in WBC was similar between the three groups investigated (Table 1). Variable levels of *F3* expression were detected in CE from FECD patients with a mean value of 61% compared to 3.6% in CE from non-FECD controls (*P*-value = 0.08) (Fig. 6c, Table 1). No expression of *PROC* was seen in CE from FECD patients nor in CE from non-FECD controls; however, scarcely detectable levels were seen in WBC in all groups tested (Table 1). High levels of *THBD* gene expression were seen in CE from FECD patients (mean = 1092%) compared to non-FECD controls (mean = 32%) (*P*-value = 0.08) (Fig. 6d, Table 1), deducing a ~ 34-fold increase of *THBD* gene expression in FECD CE. This increase was not seen in WBC since all three groups tested had similar *THBD* expression levels (mean =  ~ 6–9%) (Table 1). No expression of *F10* or *FGA* was detected in CE from FECD patients nor in CE from controls (Table 1).

Since blood clotting occurs extracellularly, we investigated if factor V interactors were expressed in adjacent tissues of the CE. The corneal stroma contains nuclear cells and is the closest tissue of the central CE, with aqueous humor residing posteriorly of the CE. For mRNA expression of *F2*, *F3*, *F10*, *PROC*, *THBD* and *FGA* gene in the corneal stroma, we analyzed corneal grafts from two non-FECD controls containing CE, Descemet’s membrane and about 100 microns of stroma. In these specimens, *F3*, *F10,* and *THBD* was expressed and no expression of *F2*, *PROC* nor *FGA* was found in these samples (Table 1).

“No reverse transcriptase” controls for CE, stroma and WBC were blank when *F5*, *AQP1*, *F2*, *F2R*, *F3*, *F10*, *PROC* and *FGA* were tested. However, since *THBD* is an intronless gene, very low levels of genomic *THBD* amplification were seen in the CE and stroma. These levels were more than eightfold lower than the lowest *THBD* expression level from a CE and stroma cDNA sample, and not considered to have a big impact on the gene expression results (Online Resource 4).

In addition, to exclude the presence of the two most common polymorphisms in *F5* and *F2* genes known to increase blood clotting and possible fibrin accumulation, all FECD patients examined for methylation pattern were genotyped for *F5* rs6025 and *F2* rs1799963. Only one FECD patient was found to be heterozygous carrier of the *F2* rs1799963G > A variant, and it was the same individual (FECD case 15) that was excluded from the analysis due to poor quality of the methylation data.

### Protein expression of F5 and THBD

**Fig. 7:**
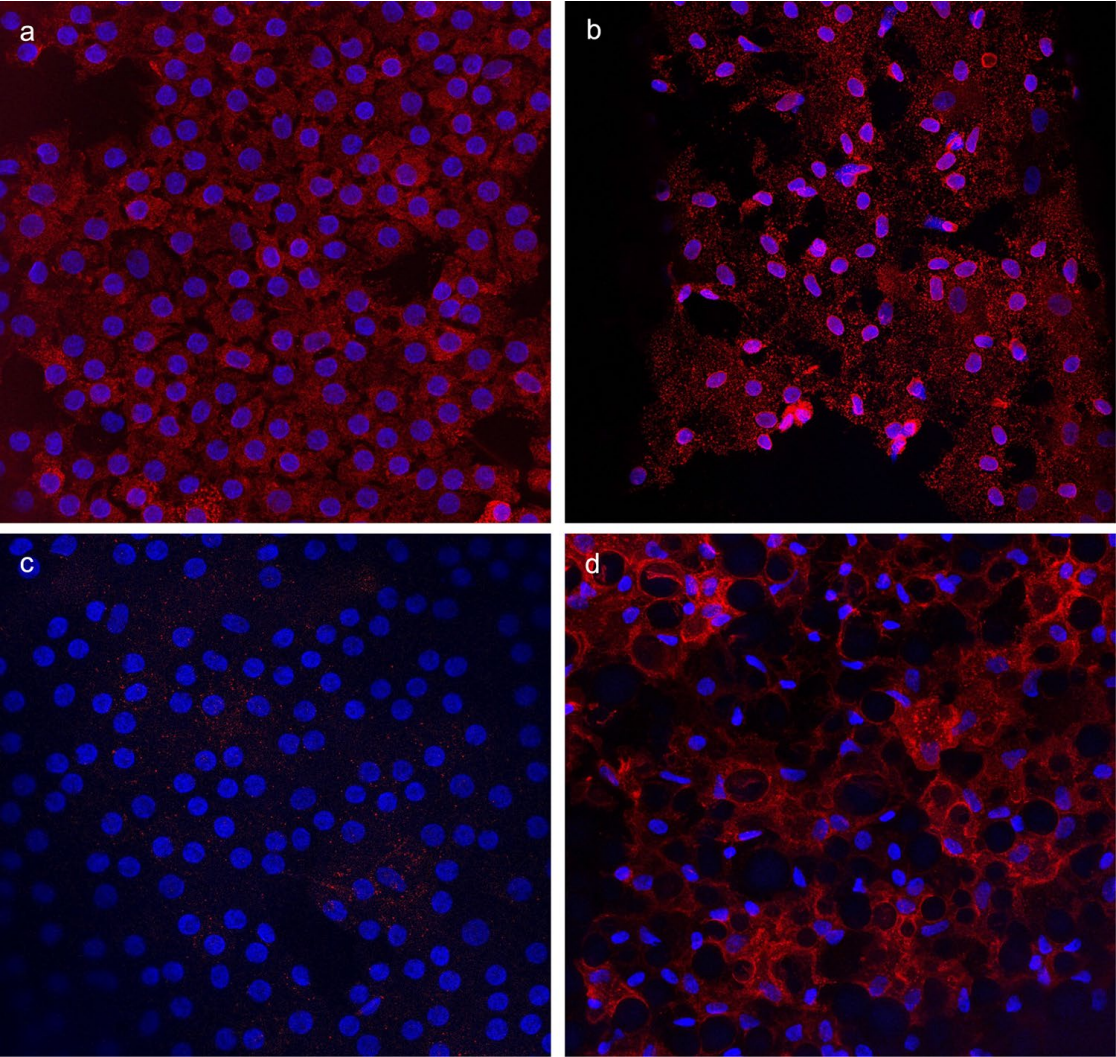
Fluorescent labeling and imaging in human corneal endothelium at 40 × magnification for (**a**) FV in non-FECD control and (**b**) FV in FECD patient and **c)** THBD in non-FECD control and (**d**) THBD in FECD patient. Nucleus are stained with DAPI (blue) and FV and THBD are shown in red. Due to highly degenerated FECD tissue, the imaging of FV and THBD presented are from two separate FECD patients

As both *F5* and *THBD* gene expression levels were significantly increased in the CE from FECD patients compared to non-FECD controls, we sought to investigate whether the protein expression also could be altered. For this, we used confocal microscopy with primary antibody against FV and THBD proteins in CE from one non-FECD control and two FECD patients. The longest CTG18.1 of the donor was 18 repeats and both FECD patients had CTG18.1 alleles > 80 repeats. Due to poorly preserved CE morphology of FECD CE, the images of FV and THBD protein analysis were taken from two separate FECD patients (Fig. 7). FV protein was seen in both the FECD patient and the non-FECD control (Fig. 7). For THBD, we found very scarce expression in the non-FECD control, while distinct protein expression of THBD was revealed in the CE from the FECD patient (Fig. 7).

## Discussion

According to the latest research, Fuchs endothelial corneal dystrophy (FECD), strongly associated with expansion of the CTG18.1 locus in the *transcription factor 4* gene, represents one of the most common trinucleotide repeat disorders [8, 18, 62, 63]. In this study we tested the hypothesis of aberrant *TCF4* methylation as a pathogenic mechanism, previously shown for trinucleotide repeat disorders such as DM1, FRDA and FRAXA [64]. The inclusion of only FECD patients with *TCF4* CTG18.1 expansion (*n* > 40 repeats) in our study allowed to investigate the relation between the *TCF4* CTG18.1 expansion and *TCF4* methylation. As a result, no DM-CpG sites were found in the *TCF4* gene among FECD patients, nor upstream or downstream of the gene. This ruled out a pathogenic role of *TCF4* methylation as a consequence of expanded CTG18.1 locus in FECD.

We also investigated global methylation in the corneal endothelium from FECD patients and non-FECD controls. Multiple DM-CpG sites were found among FECD patients, especially sites in the gene body and intergenic regions. In general, an age-dependent global hypomethylation was seen among the elderly non-FECD controls (Online Resource 1, Fig. S1). This is in line with other studies demonstrating that aging strongly correlates with decreased global methylation and, supposedly contributes to age-related diseases such as neurodegeneration, cancer, and degenerative joint diseases [37–39] though methylation changes in CE at advanced age has never been studied. One drawback of our analysis is that only two individuals were considered young (< 30 years), and no reliable statistics could be performed on the data. The global hypomethylation pattern seen in CE from non-FECD elderly donors could however reflect on the pathogenesis of FECD as a late-onset disease.

Results of the global methylation analysis revealed nine miRNAs that had more than 2 DM-CpG sites reaching the significant threshold of Δβ ± 0.2. These miRNAs have been implicated in many different diseases, such as cancer, heart defects, attention deficit, hyperactive disorder, viral infections, and oral ulcers [65–72] without indications of involvement in the FECD pathogenesis.

Based on our global methylation results, we focused on two DM-genes, the most hypermethylated (*AQP1*) and the most hypomethylated (*F5*), by conducting gene expression analysis on these two genes. Due to the limited number of cells in CE from FECD patients and non-FECD controls, CE from additional FECD patients and non-FECD controls were collected for these experiments. The *AQP1* gene was hypermethylated in the predicted promoter/enhancer region, however, the *AQP1* mRNA expression in CE from FECD patients was fluctuating, with the mean value higher in this group compared to controls. Thus, our results did not support earlier reported decreased protein levels of AQP1 in CE from FECD patients [73, 74]. AQP1 is a water channel protein that facilitates water movement across the corneal endothelium to and from the corneal stroma [61], and the shifting *AQP1* expression seen in our study may be a compensatory mechanism of the cells, to keep water movement stable when the surrounding cells have been lost.

The most striking finding in the global methylation analysis was hypomethylation of *F5* gene, which prompted us to examine the *F5* mRNA expression. We demonstrated a ~ 23-fold increase of *F5* gene expression in CE from FECD patients compared to non-FECD controls, which was in line with the loss of methylation in the predicted enhancer/promoter at position chr1:169,554,071 (GRCh37/hg19) in the FECD group. We also found that protein FV was present in both a non-FECD control and a FECD patient, though quantitative analysis was impossible to conduct. If FV levels are sustained while the mRNA is increased in FECD, there may be some sort of translational buffering that regulates FV quantity in the cells [75]. If FV levels are increased, it could contribute to FECD pathogenesis and, possibly, to the thickening of Descemet's membrane since FV is a secreted extracellular protein.

*F5* gene encodes for coagulation factor V (*F5* for gene, FV for protein), a protein mainly produced in the liver. FV is a participant of the blood clotting process characterized by both anticoagulant and procoagulant activities (Online Resource 1, Fig. S4) [76]. In addition to being part of the blood clotting cascade, prothrombin, the direct downstream target of FV can activate the prothrombin receptor; protease activated receptor 1 (PAR-1, *F2R*) [77]. PAR-1 signaling leads to increased Ca^2+^ levels and the phosphorylation of myosin light chain II (MLC) with subsequent contraction of the actin cytoskeleton, which results in gaps and breakdown of barrier integrity in vascular endothelial cells (Online Resource 1, Fig. S4) [78, 79].

FV’s involvement in blood clotting and possibly in PAR-1 signaling, impelled us to investigate the mRNA expression of six coagulation factors (*F2*, *F3*, *F10*, *FGA*, *PROC* and *THBD)* and *F2R* in the CE and stroma (not *F2R* in stroma), that are downstream or upstream targets in FV signaling [76, 77, 80].

In CE, we found no significant difference in the expression of *F2*, *F2R*, *F10*, *PROC* or *FGA* between FECD patients and non-FECD controls. In fact, we found no expression of *F10*, *PROC* nor *FGA* in CE in either group. *F3* showed an increase in expression in CE from FECD patients, although the expression was very shifting among FECD patients (4.2 to 144%, *n* = 5) compared to controls (3.5 to 3.7%, *n* = 2). The negative drawback of this data is that only two controls were used in the experiment and the numbers could therefore mirror mere coincidence and not a true difference. Moreover, no DM-CpG site was found in the *F3* gene that could explain the difference in gene expression (Online Resource 1, Fig. S5a). A more interesting finding was the distinctive ~ 34-fold increase in *THBD* expression in CE from FECD cases compared to non-FECD controls. The elevated level of *THBD* expression could not be explained by hypomethylation, since no DM-CpG sites were found in the *THBD* gene (Online Resource 1, Fig. S5b). This increase may reflect an unknown (to our knowledge) positive regulation of *THBD* expression, as a consequence of elevated *F5* mRNA expression, possibly to maintain FV inactivated in the CE. However, since *PROC* was not expressed in the CE, the direct link between THBD and FV gene regulation remains to be studied. THBD protein was highly expressed in CE from a FECD patient and scarcely present in the non-FECD control. Again, this significant difference may contribute to FECD pathogenesis and possibly to the thickening of Descemet’s membrane since THBD is a membrane-bound protein that is shed from the cell’s surface during wound healing [81]. However, these conclusions drawn from an experiment on a single individual should be interpreted cautiously and require further investigation.

In samples containing both corneal stroma and CE from non-FECD controls, we observed a consistent higher expression of *F3* (13%) and *F10* (1%) than in CE-only from non-FECD controls (4 and 0%, respectively). However, given the small number of analyzed samples (*n* = 2 versus *n* = 4), this observation should also be interpreted with some uncertainty. *THBD* had variable expression in both non-FECD groups, ranging from 2 to 16% for CE with corneal stroma and 14–49% for CE only. In WBC, no difference in gene expression was seen for any gene expression assay in any group, implying that the gene expression pattern for *AQP1*, *F5* and *THBD* in CE is tissue specific.

Both factor V mRNA and prothrombin mRNA and protein have previously been shown to be expressed in human CE [82]. Moreover, increased *F5* gene expression, placing as one of the top 20 most upregulated genes, was shown in CE with *TCF4* CTG18.1 expansion, regardless of FECD status [83]. Furthermore, RNA sequencing performed on CE from individuals with and without FECD, all carriers of *TCF4* CTG18.1 expansion did not reveal any differences in *F5* gene expression between the two groups [84]. To answer the question of whether CTG18.1 expansion might influence *F5* methylation resulting in mRNA overexpression, the inclusion of FECD patients without *TCF4* CTG18.1 expansion is certainly warranted for future DNA methylation studies.

It is known that prothrombin and fibrinogen are constituents of the aqueous humor in the eye, the liquid formed from filtered blood in the ciliary body [85, 86]. We have, however, not investigated the presence of factor V interacting proteins extracellularly of the CE. In regard to our findings, the aqueous humor and corneal stroma could be valuable components to study blood clotting proteins in FECD patients.

Lack of the *TCF4* genotype and different filtering methods in previous FECD methylation studies could be one of the reasons why there is not much agreement in our results compared to Khuc et al*.* [36] and Pan et al. [25] who used Illumina 450 K methylation array on samples with unknown *TCF4* CTG18.1 genotype. We applied a different method to calculate significant changes between FECD and controls; β-values versus M-values and an absolute difference versus linear model, which could also bring the dissimilarities. Moreover, 90% (18 out of 20) of Khuc et al*.* [36] top 20 CpG sites and 35% (7 out of 20) of Pan et al. [25] top 20 CpG sites were filtered out in our pre-analysis as either meQTL, SNP, aligned to multiple loci or background noise. We did however analyzed the previous dataset (GEO GSE94462) according to our method, and found that the same probe in *AQP1* corroborated with our results. The probe cg13122356 in *F5* does not exist on the Illumina 450 K methylation array and could therefore not be analyzed.

We also acknowledge that it is challenging to filter out all possible CpG sites that may be affected by meQTLs. However, in order to study non-genetic epigenetic changes, we have applied best practice to filter out regions denoted as meQTL with current knowledge.

## Conclusions

In conclusion, significant global methylation changes in corneal endothelium from FECD patients with *TCF4* CTG18.1 repeat expansion are an indisputable fact. Hypomethylation of coagulation factor V and the significant increase of its and thrombomodulin’s mRNA expression might reveal novel mechanisms in the appearance and/or progress of FECD. At the same time, currently unknown functions of factor V, besides its important role in the coagulation cascade, can be discovered. Notably, that in FECD no methylation changes of CpGs located in the key gene *TCF4* were detected, which rules out hypermethylation as a pathogenic disease mechanism.

## Supplementary Information

Below is the link to the electronic supplementary material.

Supplementary file1 (PDF 496 KB)

Supplementary file2 (XLSX 1655 KB)

Supplementary file3 (PDF 2852 KB)

Supplementary file4 (XLSX 50 KB)

## Data Availability

The datasets generated and/or analyzed during the current study are available in the National Center for Biotechnology Information (NCBI) Gene Expression Omnibus (GEO) repository at https://www.ncbi.nlm.nih.gov/geo under the accession number: GSE198917.
