# Supplementary material for: DNA methylation changes and increased mRNA expression of coagulation proteins, factor V and thrombomodulin in Fuchs endothelial corneal dystrophy
Source: Cell Mol Life Sci. 2023 Feb 11;80(3):62. doi: 10.1007/s00018-023-04714-x (PMC9922242; doi:10.1007/s00018-023-04714-x)
Supplement: Supplementary file 1 — Supplementary file1 (PDF 496 KB) [file 18_2023_4714_MOESM1_ESM.pdf]

DNA methylation changes and increased mRNA expression of coagulation proteins, Factor V and Thrombomodulin in Fuchs endothelial corneal dystrophy in "Cellular and Molecular Life Sciences" by
Westin IM, Landfors M, Giannopoulos A, Viberg A, Osterman P, Byström B, Degerman S, Golovleva I*.
*Correspondence to Irina Golovleva, Clinical Genetics, University Hospital, SE 901 85, Umeå, Sweden. E-mail_ irina.golovleva@umu.se

Supplementary Information

Online Resource 1

Supplementary Table S1.

FECD patients and non-FECD controls in the study

	FECD methylation CE (*n* = 16)	Controls methylation CE (*n* = 11)	FECD gene expression CE (*n* = 5)	FECD gene expression WBC (*n* = 14)	Controls gene expression CE and WBC (*n* = 4 and 1)
**Age in years**					
median age (IQR)	72.5 (16.5)	76 (27)	76 (18)	72 (11)	59 (36)
range age	59 to 84	26 to 96	63 to 85	59 to 85	26 to 78
**Sex**					
female	12 (75%)	6 (55%)	1 (20%)	8 (57%)	4 (80%)
male	4 (25%	5 (45%)	4 (80%)	6 (43%)	1 (20%)
**Cataract surgery**					
yes	16 (100%)	3 (27%)	5 (100%)	10 (71%)	1 (20%)
no	0 (0%)	8 (73%)	0 (0%)	-	3 (60%)
unknown	-	-	-	4 (29%)	1 (20%)
***TCF4* CTG18.1**					
> 40 repeats	16 (100%)	0 (0%)	5 (100%)	10 (71%)	0 (0%)
median repeats (IQR)	94 (15.5)	16 (8)	87 (26)	84 (78)	18 (3.5)
range repeats	52 to > 125	13 to 25	71 to 105	13 to > 125	18 to 25

CE – cornea endothelium, WBC – white blood cells.

Westin IM, Landfors M, Giannopoulos A, Viberg A, Osterman P, Byström B, Degerman S, Golovleva I*.
*Correspondence to Irina Golovleva, Clinical Genetics, University Hospital, SE 901 85, Umeå, Sweden. E-mail_ irina.golovleva@umu.se

Supplementary Figure S1

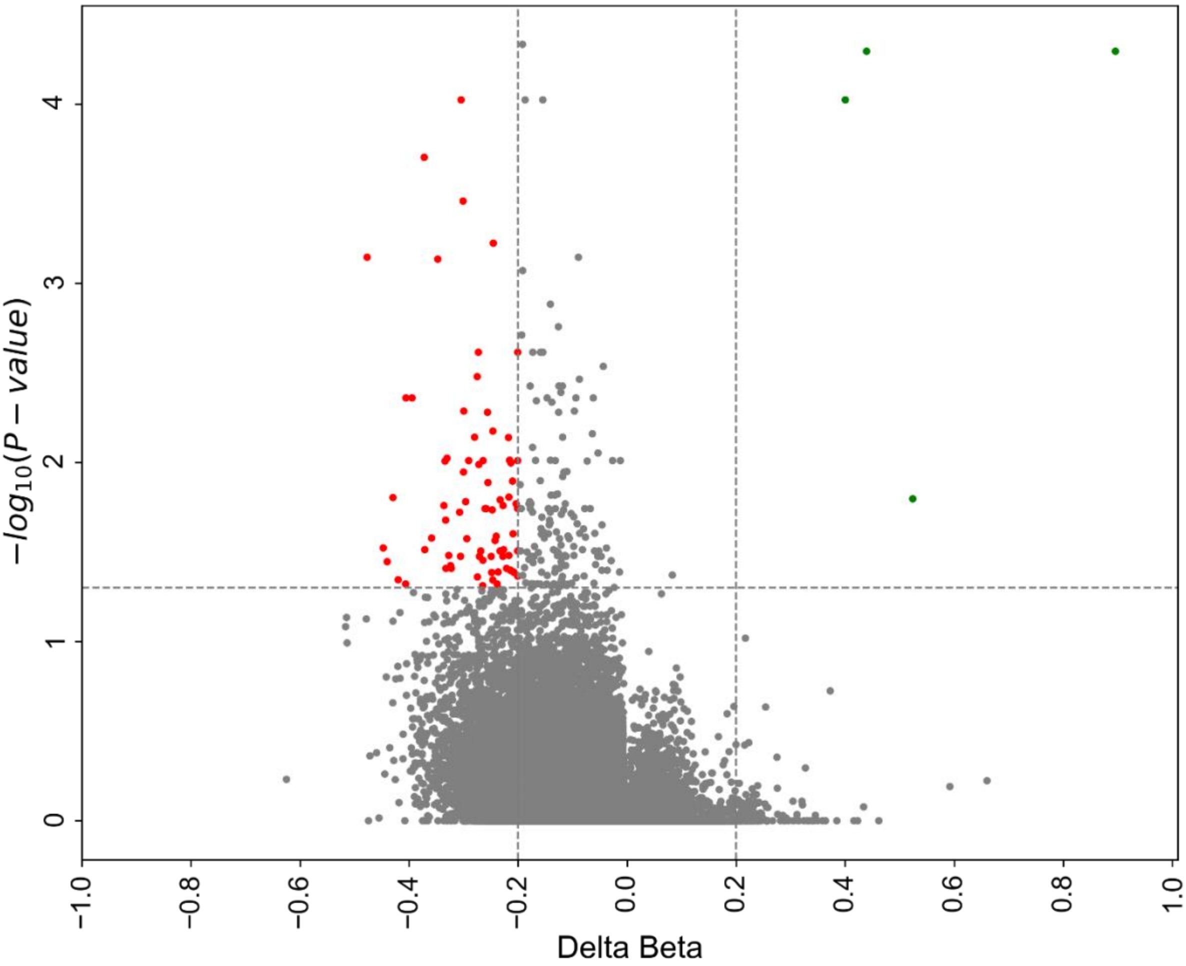

Supplementary Fig. S1 Volcano plot showing statistical significance and delta beta (Δβ) values of individual probes for elderly non-FECD controls (> 57 years) compared to younger non-FECD controls (< 30 years). The red dots indicate CpG sites with Δβ values below -0.20 and green dots indicate CpG sites with Δβ values above 0.20. The horizontal dotted line shows threshold significance of adjusted p-value 0.05 and the vertical dotted line displays thresholds of Δβ ± 0.2

DNA methylation changes and increased mRNA expression of coagulation proteins, Factor V and Thrombomodulin in Fuchs endothelial corneal dystrophy in "Cellular and Molecular Life Sciences" by
Westin IM, Landfors M, Giannopoulos A, Viberg A, Osterman P, Byström B, Degerman S, Golovleva I*.
*Correspondence to Irina Golovleva, Clinical Genetics, University Hospital, SE 901 85, Umeå, Sweden. E-mail_ irina.golovleva@umu.se

Supplementary Figure S2

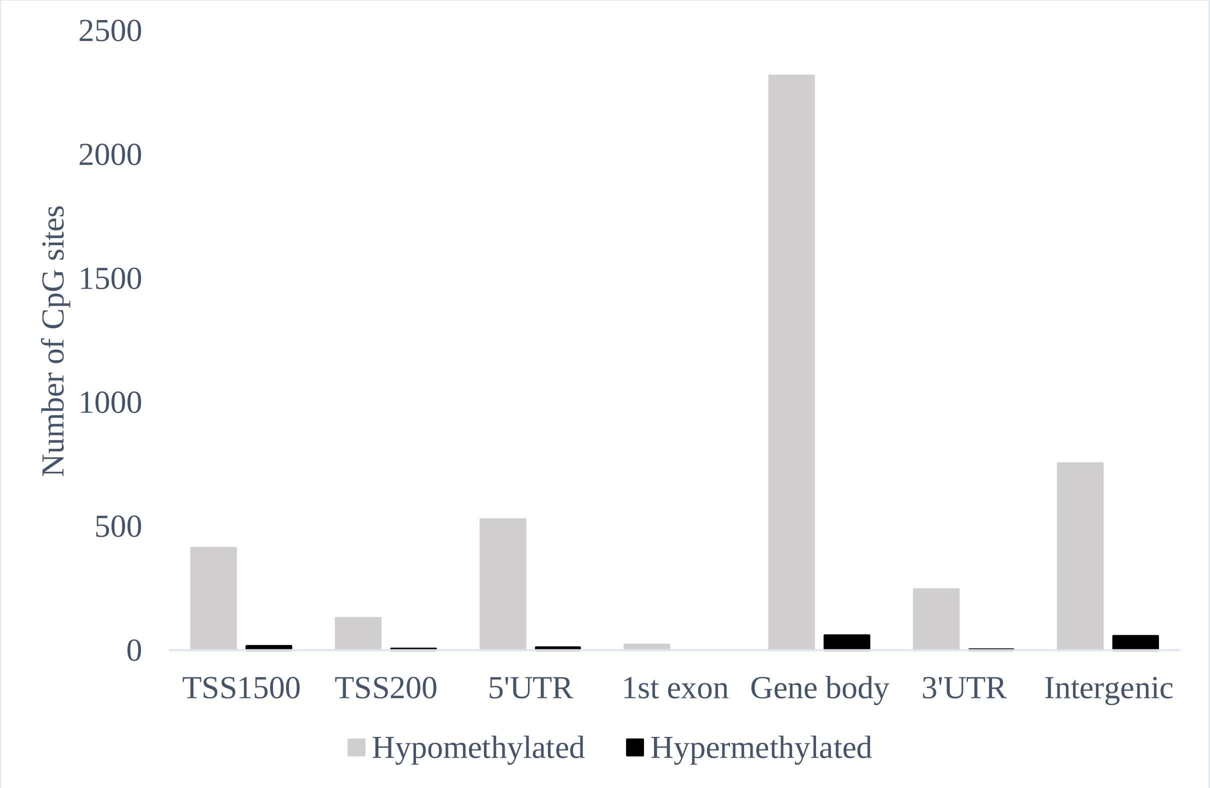

Supplementary Fig. S2 Number of differently methylated CpG sites (Δβ ± 0.2) between young (< 30 years) and elderly non-FECD controls (> 57 years), grouped by targeted genomic region. Grey stacks display hypomethylated CpG sites and black stacks display hypermethylated CpG sites. TSS1500 – 1500 bp from transcription start site, TSS200 - 200 bp from transcription start site

.

Supplementary Figure S3

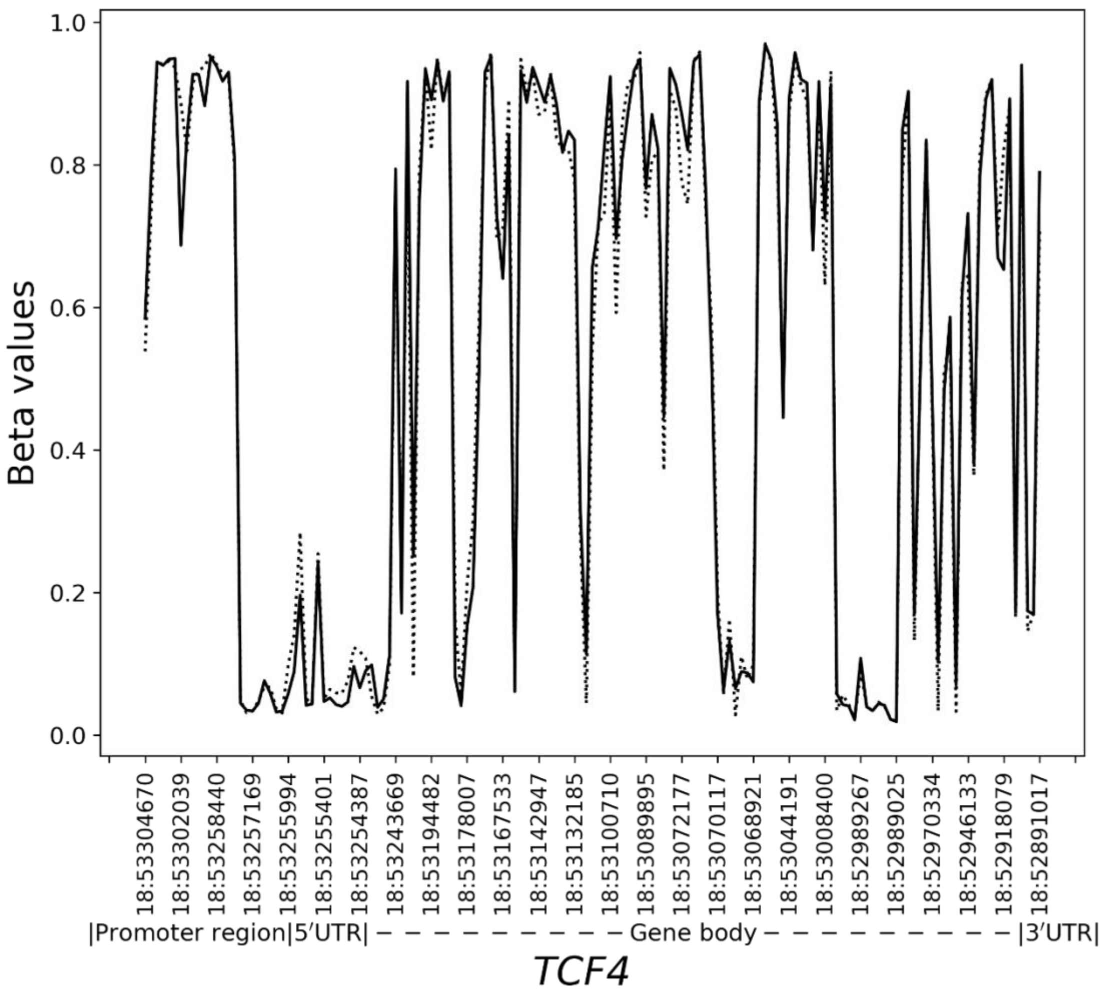

Supplementary Fig. S3 Mean methylation level (β) in corneal endothelium from non-FECD controls (dotted line) and FECD patients (continuous line) in the *TCF4* gene. Gene region annotation according to the MANE select and canonical transcript NM_001083962.2, known as TCF4-B+. Genomic positions are according to genome build GRCh37/hg19 and are shown for every 6th CpG position

DNA methylation changes and increased mRNA expression of coagulation proteins, Factor V and Thrombomodulin in Fuchs endothelial corneal dystrophy in "Cellular and Molecular Life Sciences" by
Westin IM, Landfors M, Giannopoulos A, Viberg A, Osterman P, Byström B, Degerman S, Golovleva I*.
*Correspondence to Irina Golovleva, Clinical Genetics, University Hospital, SE 901 85, Umeå, Sweden. E-mail_ irina.golovleva@umu.se

Supplementary Figure S4

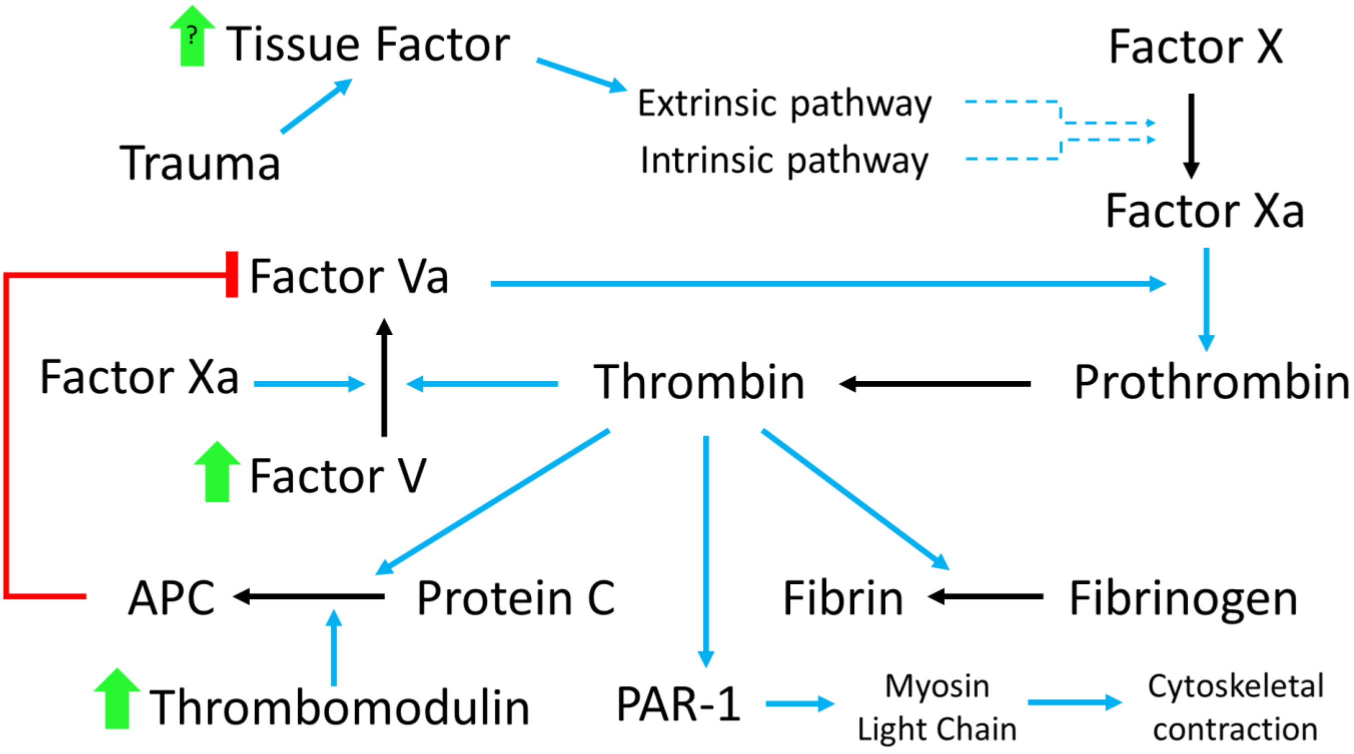

Supplementary Fig. S4 Interacting proteins and signaling pathway for Factor V (FV-protein, *F5*-gene). All factors displayed, except for myosin light chain were investigated for mRNA levels in corneal endothelium and white blood cells. Blue lines indicate interaction, black lines indicate activation, red line indicate inactivation and green arrows indicate elevated mRNA levels in CE from FECD patients in the present study

DNA methylation changes and increased mRNA expression of coagulation proteins, Factor V and Thrombomodulin in Fuchs endothelial corneal dystrophy in “Cellular and Molecular Life Sciences” by
Westin IM, Landfors M, Giannopoulos A, Viberg A, Osterman P, Byström B, Degerman S, Golovleva I*.
*Correspondence to Irina Golovleva, Clinical Genetics, University Hospital, SE 901 85, Umeå, Sweden. E-mail_ irina.golovleva@umu.se

Supplementary Figure S5

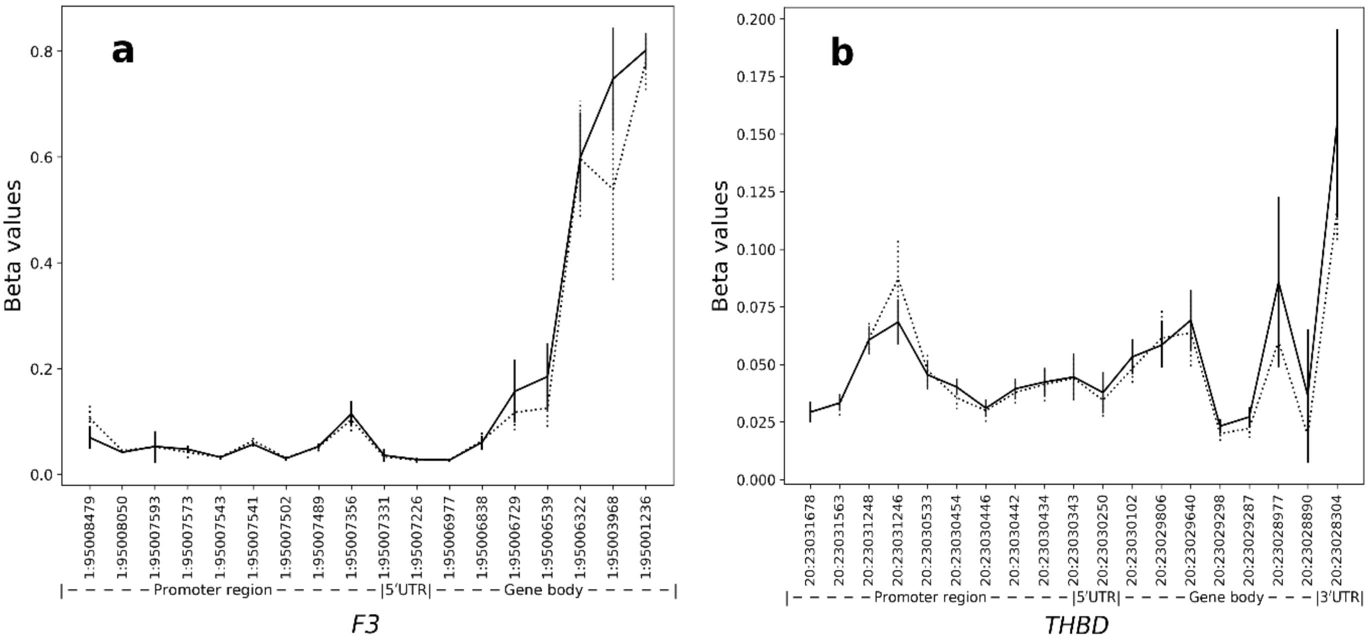

Supplementary Fig. S5 Mean methylation level (β) in corneal endothelium from non-FECD controls (dotted line) and FECD patients (continuous line) in the **a**) *F3* gene and **b**) *THBD* gene. Gene region annotation according to the MANE select and canonical transcripts NM_001993.5 (*F3*) and NM_000361.3 (*THBD*). Vertical lines show standard deviation at each CpG site. Genomic positions are according to genome build GRCh37/hg19
