# Supplementary material for: DNA methylation changes and increased mRNA expression of coagulation proteins, factor V and thrombomodulin in Fuchs endothelial corneal dystrophy
Source: Cell Mol Life Sci. 2023 Feb 11;80(3):62. doi: 10.1007/s00018-023-04714-x (PMC9922242; doi:10.1007/s00018-023-04714-x)

DNA methylation changes and increased mRNA expression of coagulation proteins, Factor V and Thrombomodulin in Fuchs endothelial corneal dystrophy in "Cellular and Molecular Life Sciences" by Westin IM, Landfors M, Giannopoulos A, Viberg A, Osterman P, Byström B, Degerman S, Golovleva I*.
*Correspondence to Irina Golovleva, Clinical Genetics, University Hospital, SE 901 85, Umeå, Sweden. E-mail_ irina.golovleva@umu.se

Supplementary Information

Online Resource 3

miRNAs in Fuchs endothelial corneal dystrophy with ≥ 2 DM-CpGs

For probes in our dataset that had ≥ 2 CpG sites above the significant threshold of Δβ value ± 0.2, and that are associated with miRNAs, the miRNA genes in our dataset were plotted. Figures show mean methylation level (β) in the corneal endothelium from non-FECD controls (dotted line) and FECD patients (continuous line). Vertical lines show standard deviation at each CpG site (due to resolution, this was not possible for genes containing many CpGs). All genes are plotted in 5'→3' direction. Genomic positions are according to genome build GRCh37/hg19

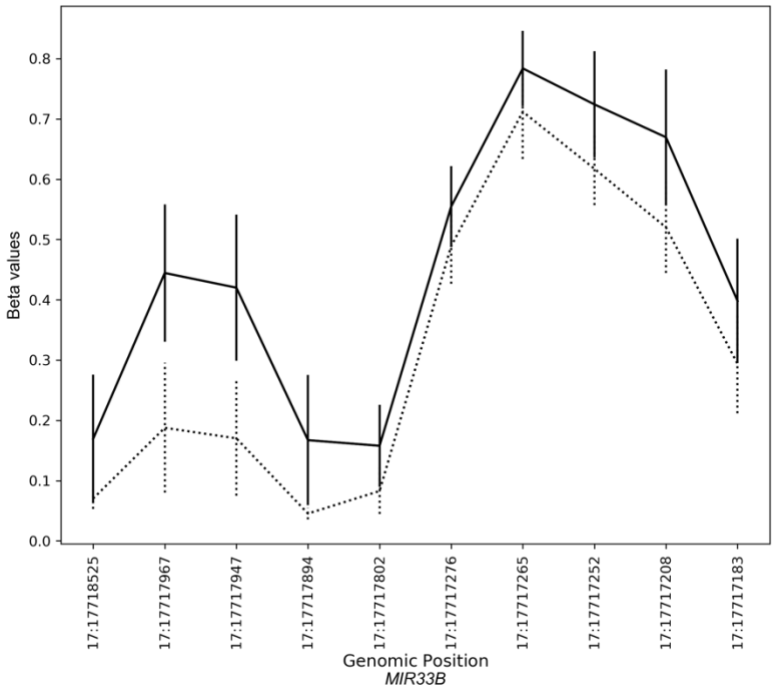

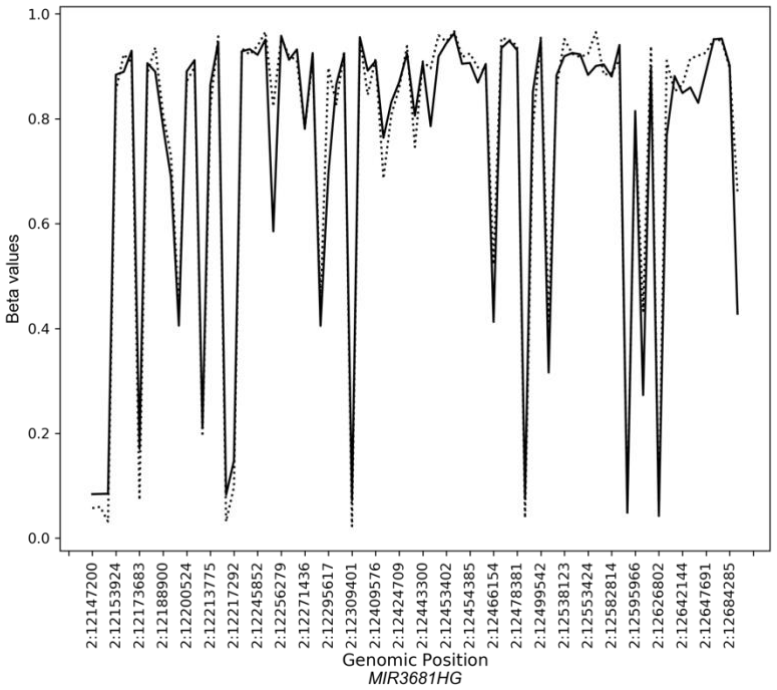

DNA methylation changes and increased mRNA expression of coagulation proteins, Factor V and Thrombomodulin in Fuchs endothelial corneal dystrophy in “Cellular and Molecular Life Sciences” by Westin IM, Landfors M, Giannopoulos A, Viberg A, Osterman P, Byström B, Degerman S, Golovleva I*.

*Correspondence to Irina Golovleva, Clinical Genetics, University Hospital, SE 901 85, Umeå, Sweden. E-mail_ irina.golovleva@umu.se

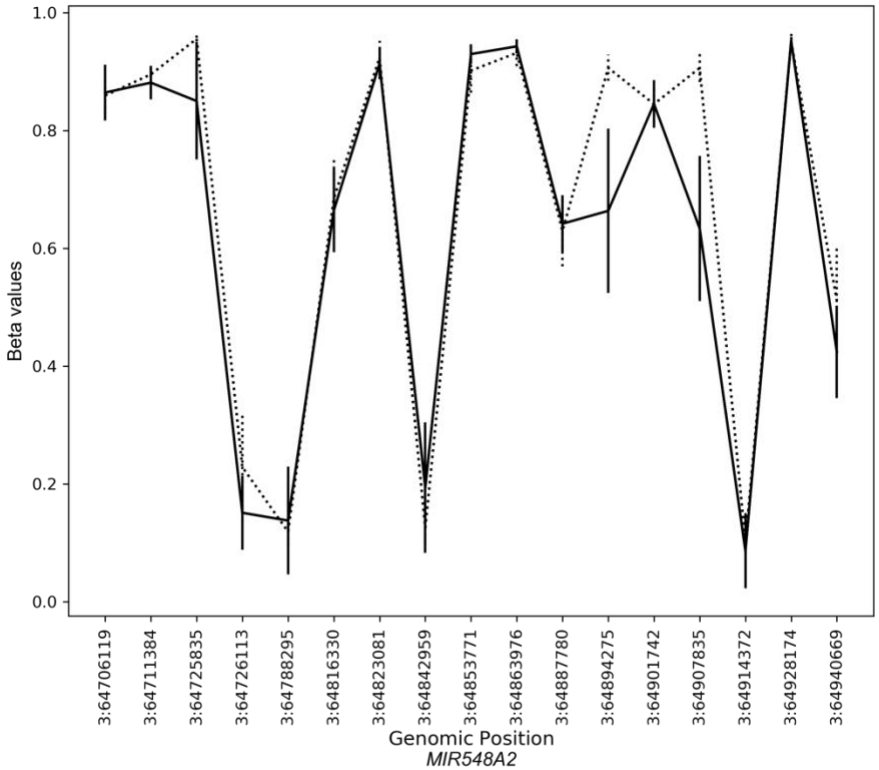

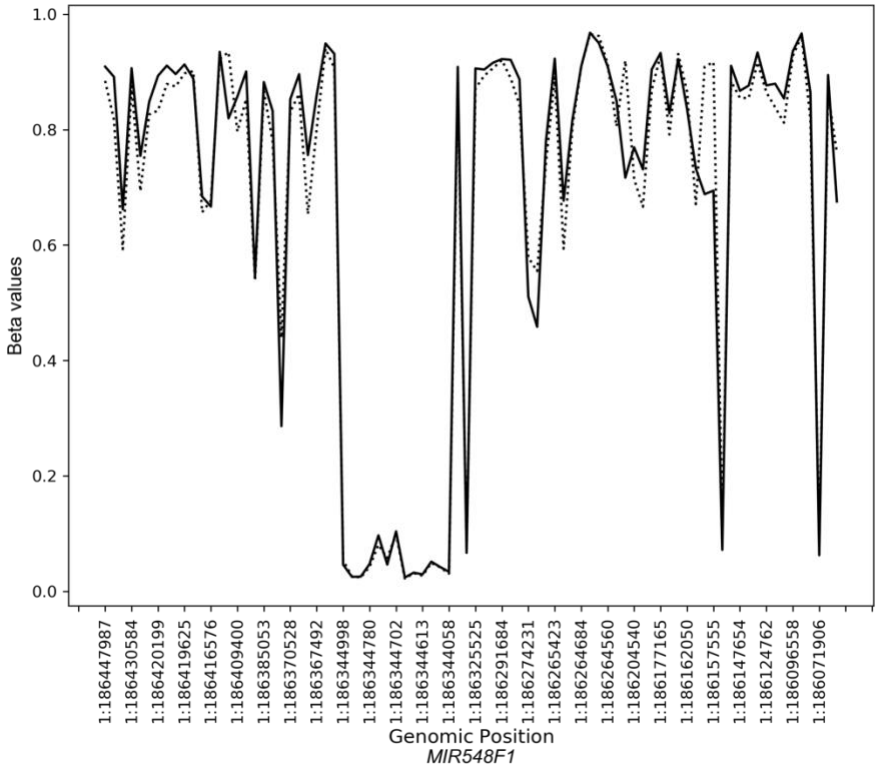

DNA methylation changes and increased mRNA expression of coagulation proteins, Factor V and Thrombomodulin in Fuchs endothelial corneal dystrophy in “Cellular and Molecular Life Sciences” by Westin IM, Landfors M, Giannopoulos A, Viberg A, Osterman P, Byström B, Degerman S, Golovleva I*.

*Correspondence to Irina Golovleva, Clinical Genetics, University Hospital, SE 901 85, Umeå, Sweden. E-mail_ irina.golovleva@umu.se

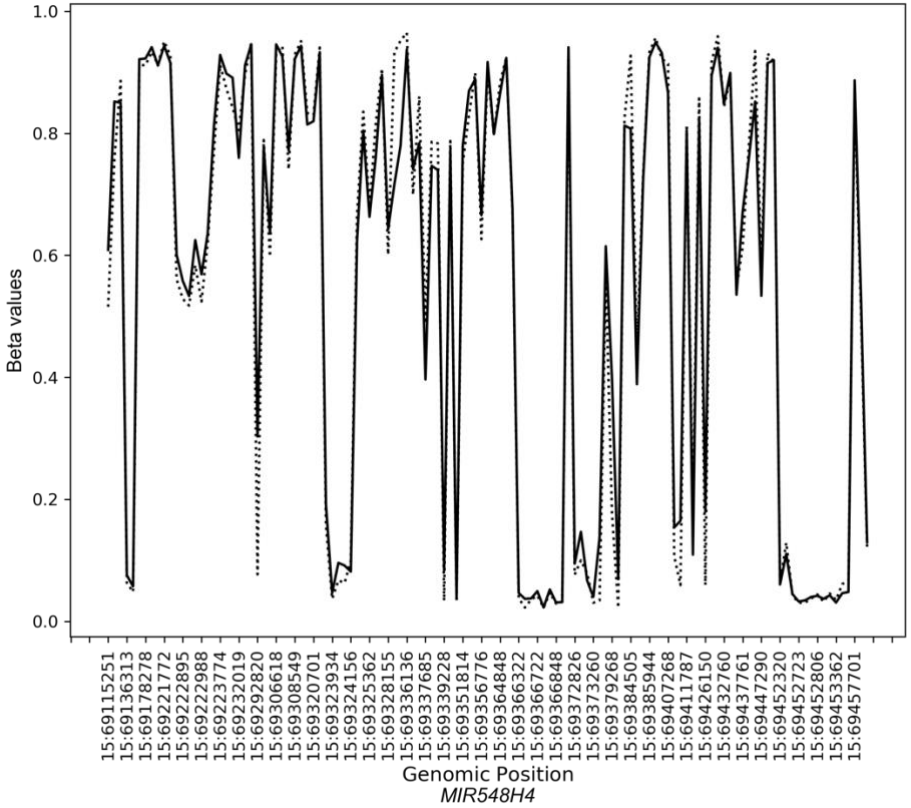

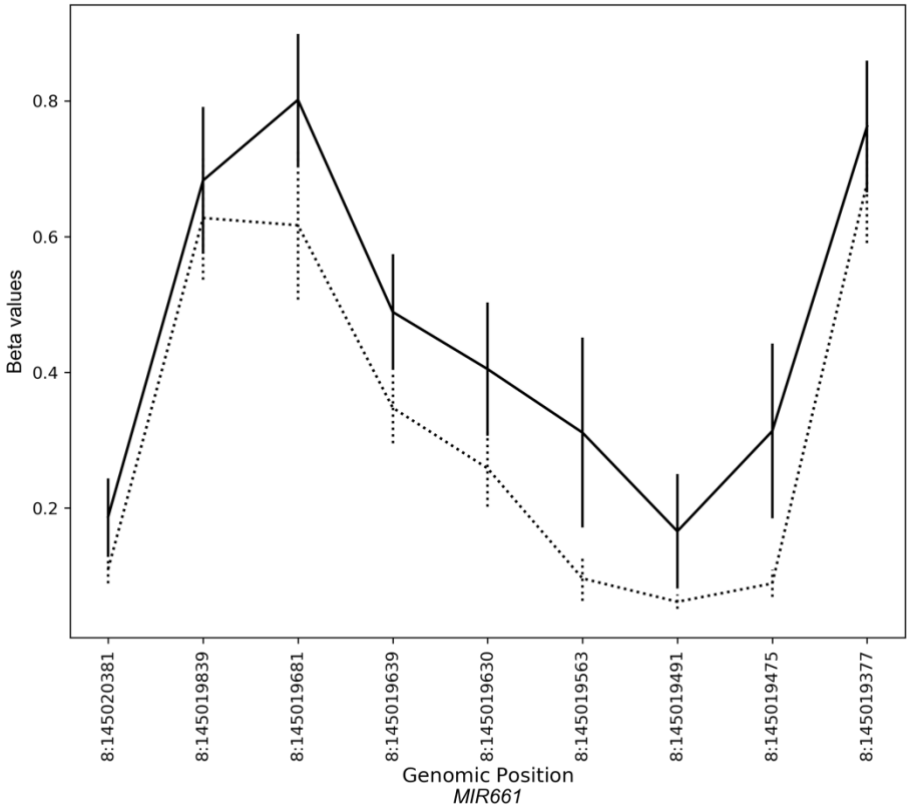

DNA methylation changes and increased mRNA expression of coagulation proteins, Factor V and Thrombomodulin in Fuchs endothelial corneal dystrophy in “Cellular and Molecular Life Sciences” by Westin IM, Landfors M, Giannopoulos A, Viberg A, Osterman P, Byström B, Degerman S, Golovleva I*.

*Correspondence to Irina Golovleva, Clinical Genetics, University Hospital, SE 901 85, Umeå, Sweden. E-mail_ irina.golovleva@umu.se

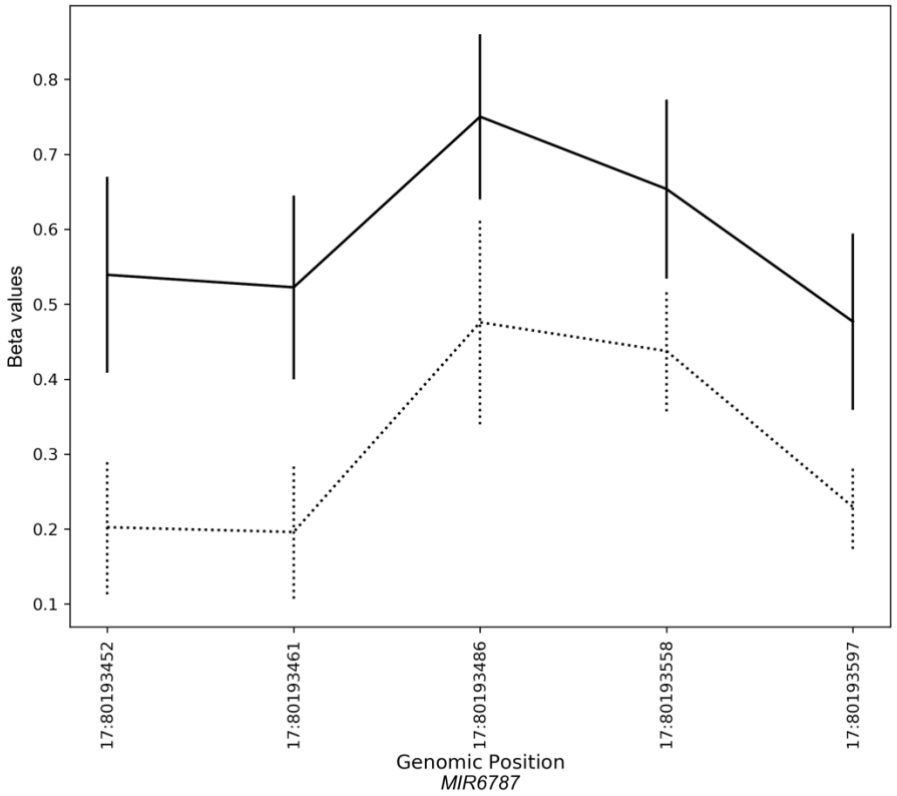

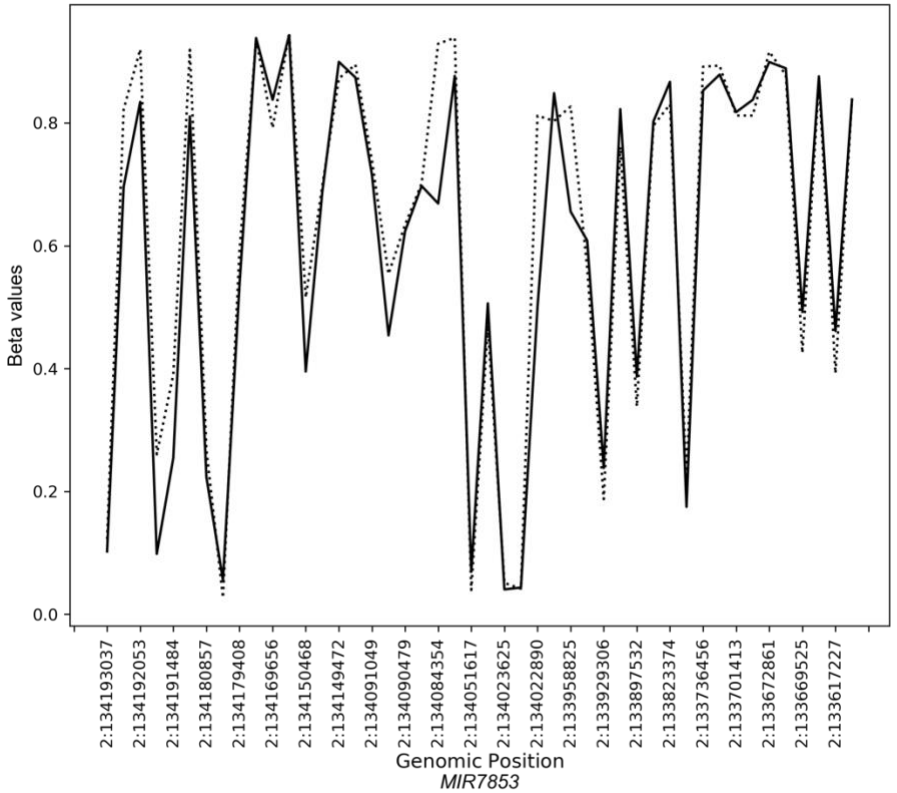

DNA methylation changes and increased mRNA expression of coagulation proteins, Factor V and Thrombomodulin in Fuchs endothelial corneal dystrophy in “Cellular and Molecular Life Sciences” by Westin IM, Landfors M, Giannopoulos A, Viberg A, Osterman P, Byström B, Degerman S, Golovleva I*.
*Correspondence to Irina Golovleva, Clinical Genetics, University Hospital, SE 901 85, Umeå, Sweden. E-mail_ irina.golovleva@umu.se

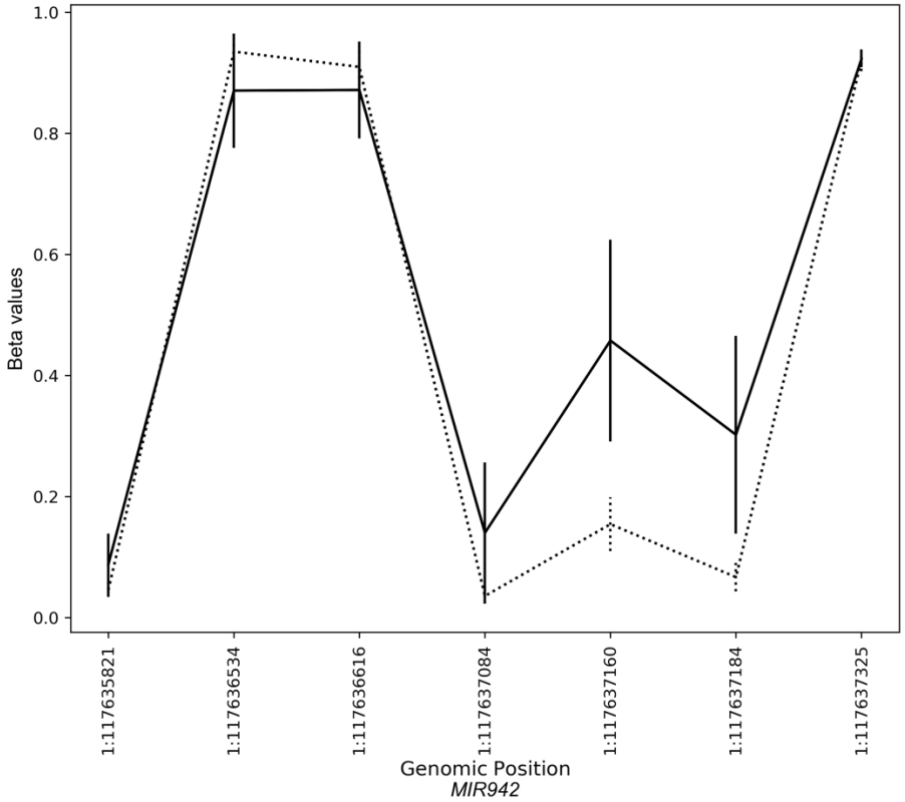

DNA methylation changes and increased mRNA expression of coagulation proteins, Factor V and Thrombomodulin in Fuchs endothelial corneal dystrophy in "Cellular and Molecular Life Sciences" by Westin IM, Landfors M, Giannopoulos A, Viberg A, Osterman P, Byström B, Degerman S, Golovleva I*.
*Correspondence to Irina Golovleva, Clinical Genetics, University Hospital, SE 901 85, Umeå, Sweden. E-mail_ irina.golovleva@umu.se

DNA methylation levels of genes associated with FECD

Figures show mean methylation level (β) in the corneal endothelium from non-FECD controls (dotted line) and FECD patients (continuous line). Vertical lines show standard deviation at each CpG site (due to resolution, this was not possible for genes containing many CpGs). All genes are plotted in 5'→3' direction. Genomic positions are according to genome build GRCh37/hg19

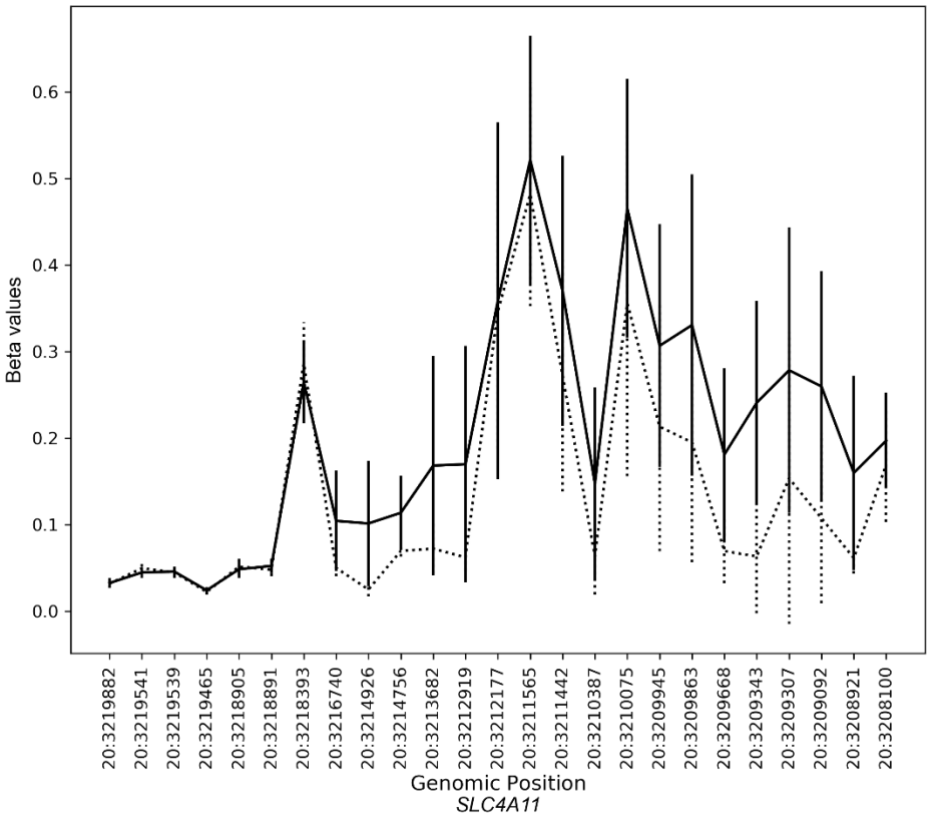

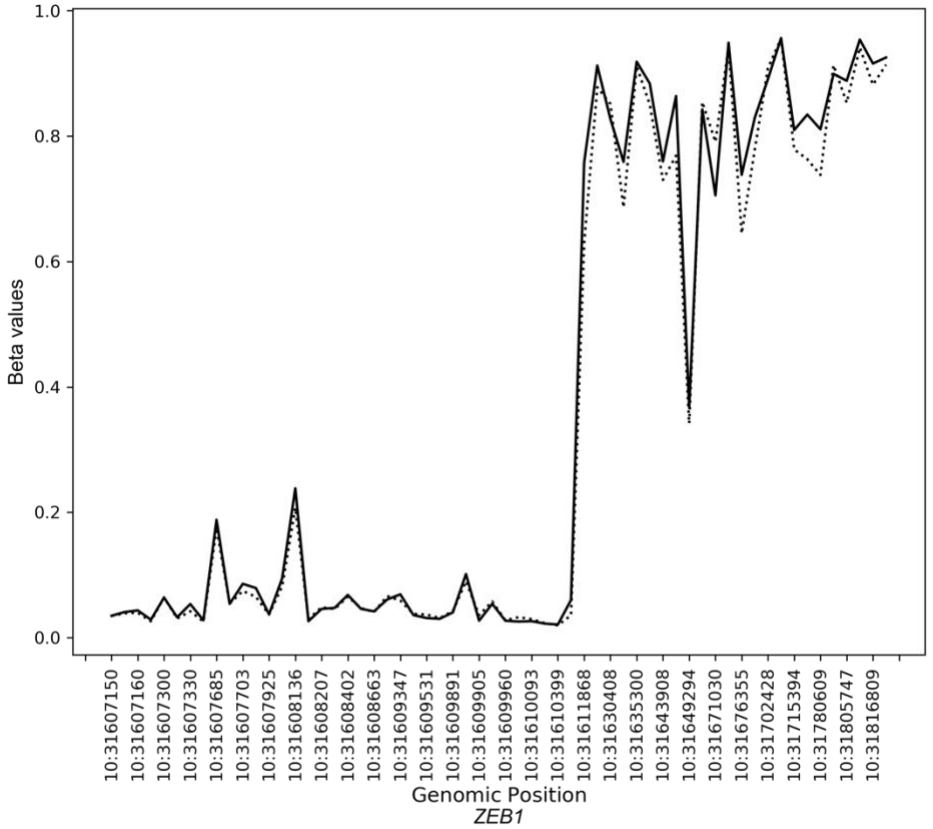

DNA methylation changes and increased mRNA expression of coagulation proteins, Factor V and Thrombomodulin in Fuchs endothelial corneal dystrophy in “Cellular and Molecular Life Sciences” by Westin IM, Landfors M, Giannopoulos A, Viberg A, Osterman P, Byström B, Degerman S, Golovleva I*.
*Correspondence to Irina Golovleva, Clinical Genetics, University Hospital, SE 901 85, Umeå, Sweden. E-mail_ irina.golovleva@umu.se

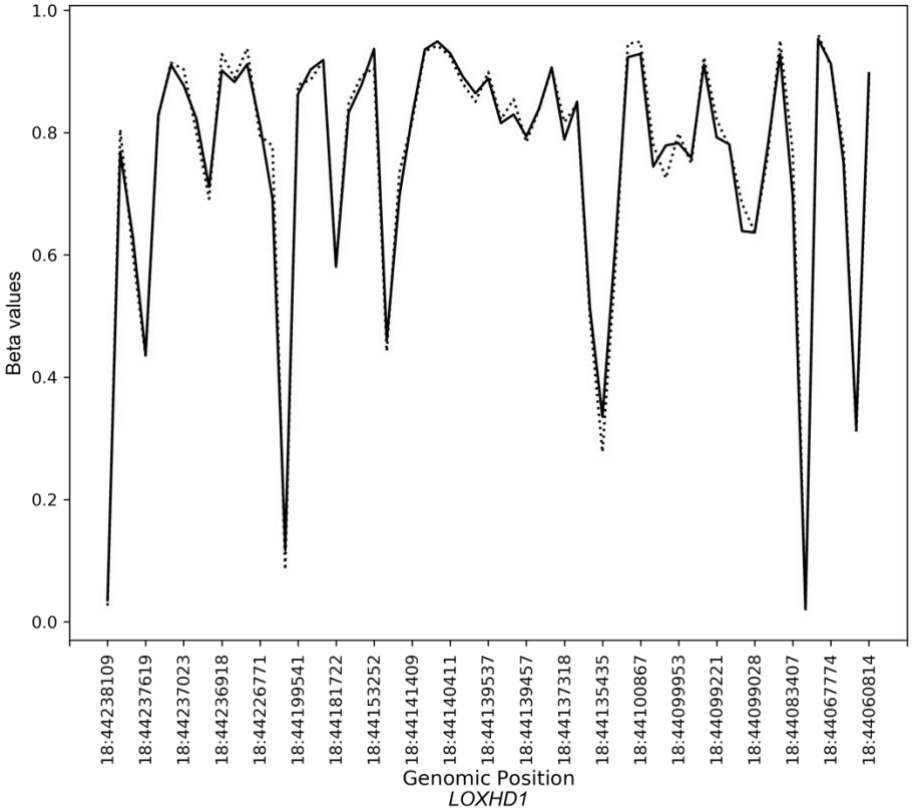

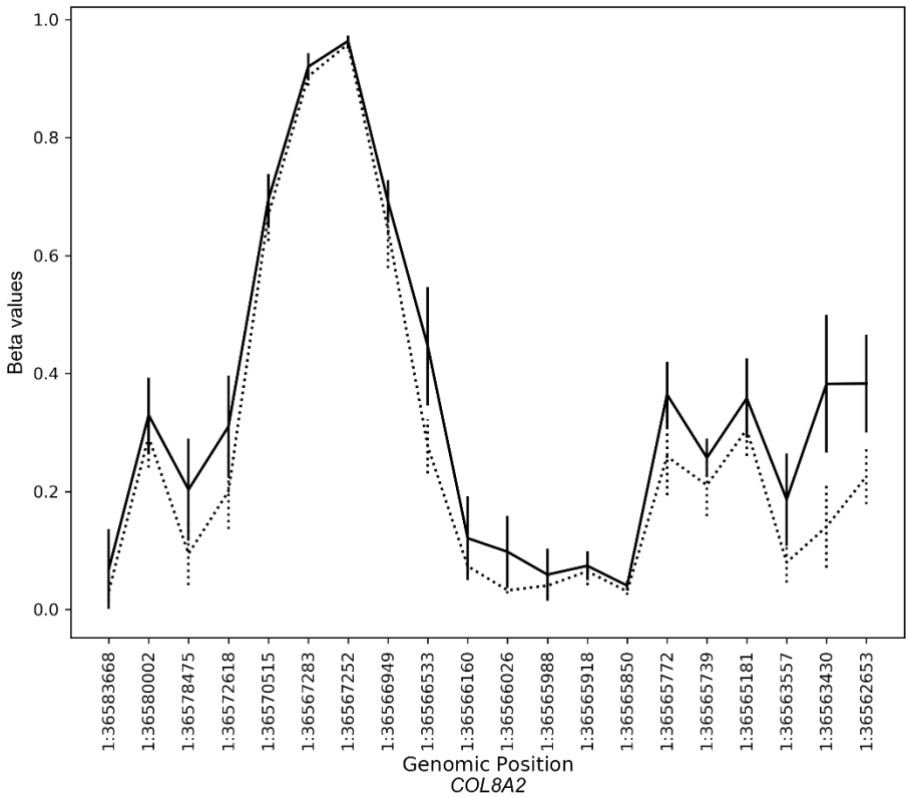

DNA methylation changes and increased mRNA expression of coagulation proteins, Factor V and Thrombomodulin in Fuchs endothelial corneal dystrophy in “Cellular and Molecular Life Sciences” by Westin IM, Landfors M, Giannopoulos A, Viberg A, Osterman P, Byström B, Degerman S, Golovleva I*.

*Correspondence to Irina Golovleva, Clinical Genetics, University Hospital, SE 901 85, Umeå, Sweden. E-mail_ irina.golovleva@umu.se

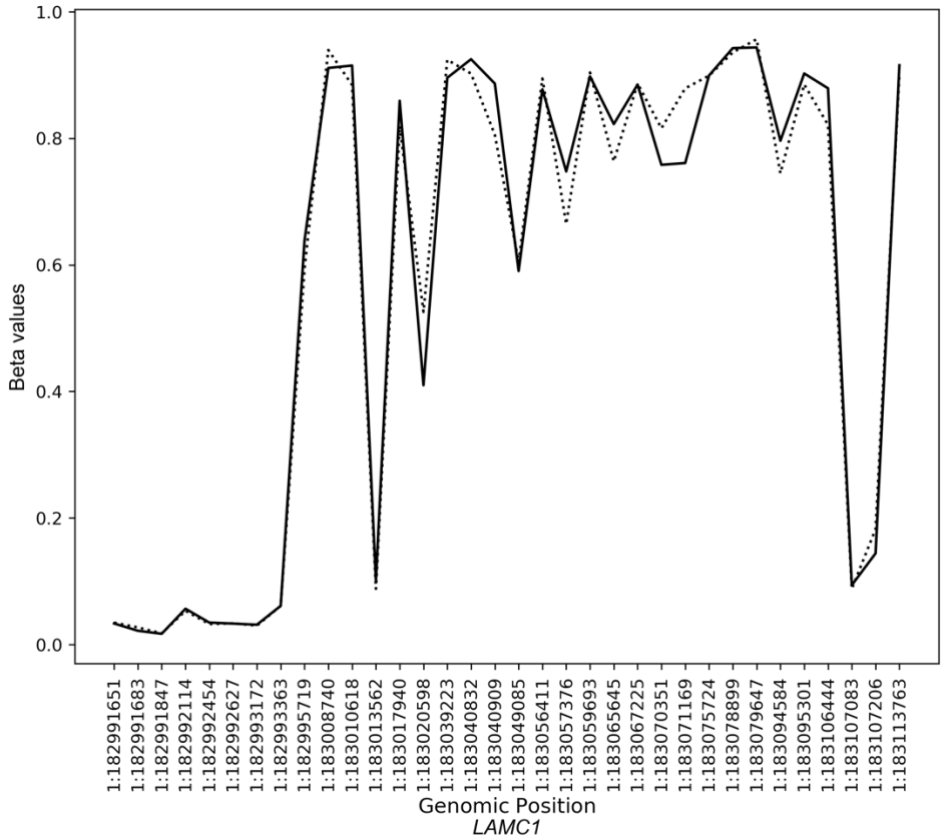

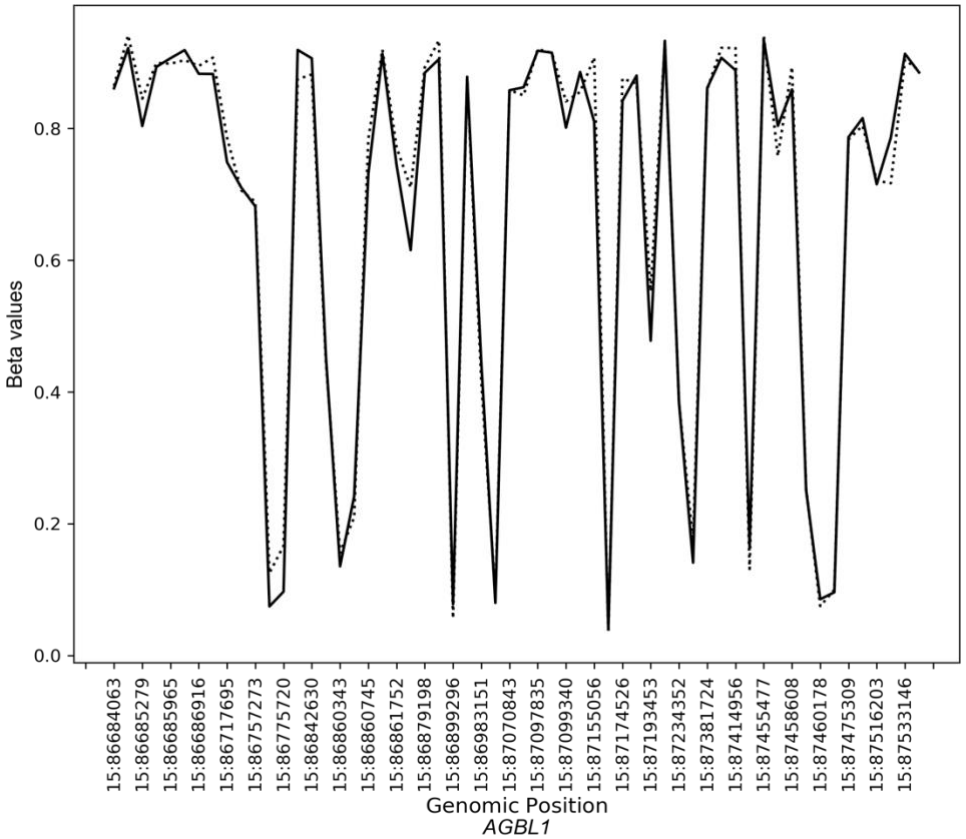

DNA methylation changes and increased mRNA expression of coagulation proteins, Factor V and Thrombomodulin in Fuchs endothelial corneal dystrophy in “Cellular and Molecular Life Sciences” by Westin IM, Landfors M, Giannopoulos A, Viberg A, Osterman P, Byström B, Degerman S, Golovleva I*.

*Correspondence to Irina Golovleva, Clinical Genetics, University Hospital, SE 901 85, Umeå, Sweden. E-mail_ irina.golovleva@umu.se

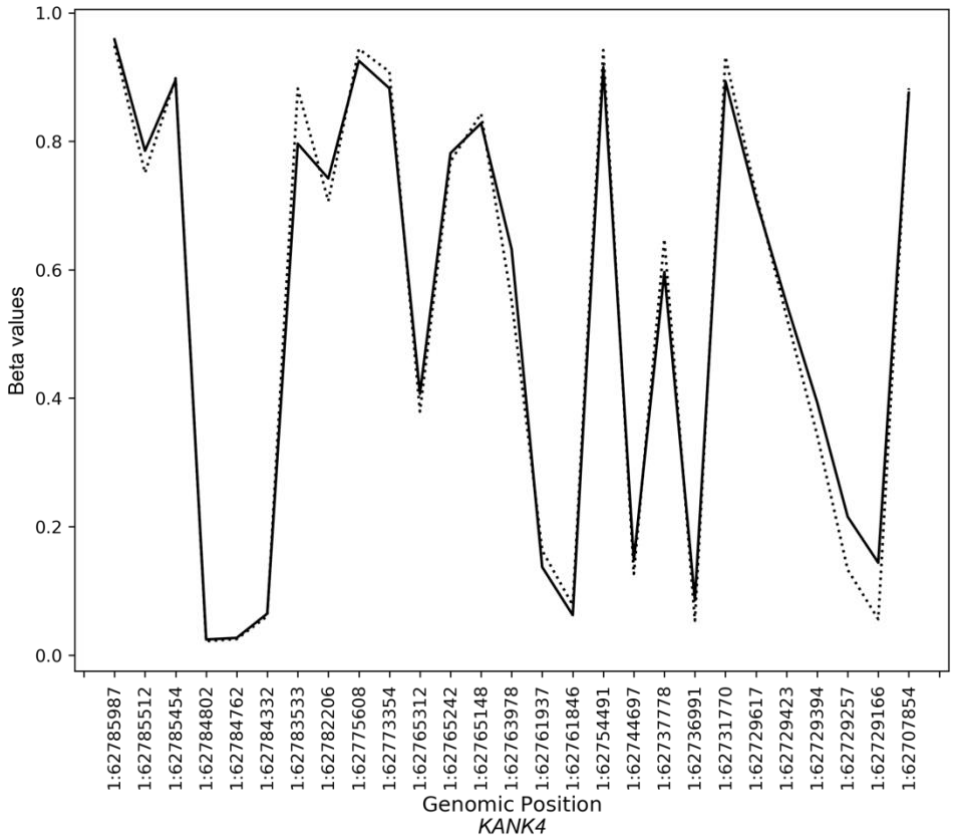

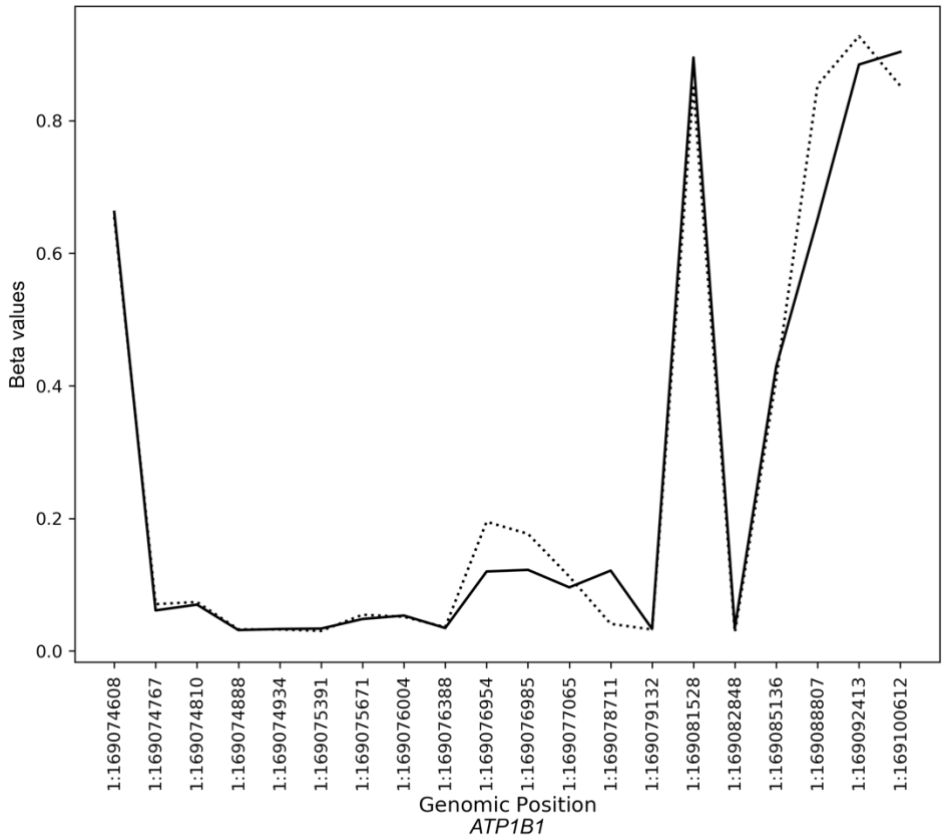

DNA methylation changes and increased mRNA expression of coagulation proteins, Factor V and Thrombomodulin in Fuchs endothelial corneal dystrophy in “Cellular and Molecular Life Sciences” by Westin IM, Landfors M, Giannopoulos A, Viberg A, Osterman P, Byström B, Degerman S, Golovleva I*.
*Correspondence to Irina Golovleva, Clinical Genetics, University Hospital, SE 901 85, Umeå, Sweden. E-mail_ irina.golovleva@umu.se

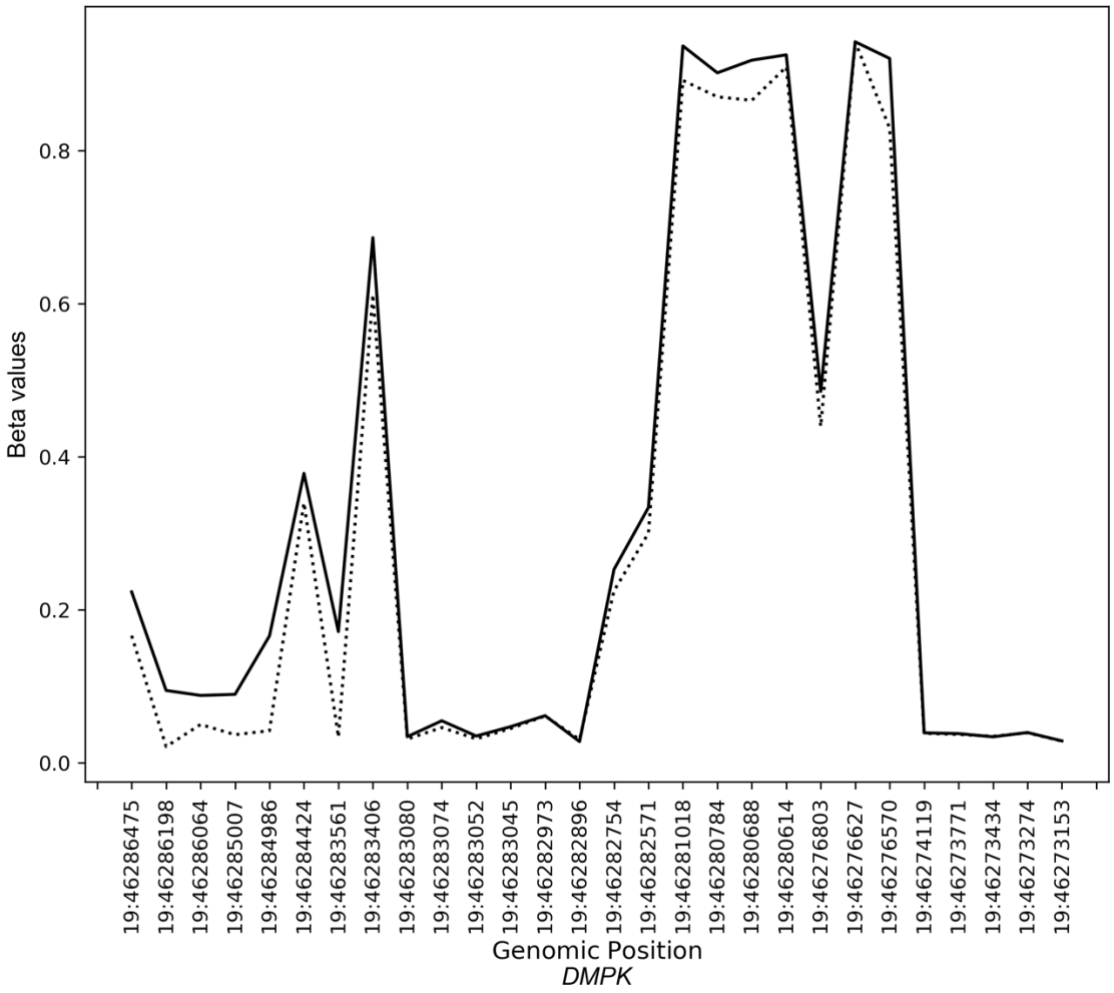

DNA methylation changes and increased mRNA expression of coagulation proteins, Factor V and Thrombomodulin in Fuchs endothelial corneal dystrophy in "Cellular and Molecular Life Sciences" by Westin IM, Landfors M, Giannopoulos A, Viberg A, Osterman P, Byström B, Degerman S, Golovleva I*.
*Correspondence to Irina Golovleva, Clinical Genetics, University Hospital, SE 901 85, Umeå, Sweden. E-mail_ irina.golovleva@umu.se

DNA methylation levels of genes containing CpGs overlapping with top 20 CpGs in Khuc *et al*. (2017)

For probes in our dataset that overlap with top 20 probes in Khuc et al. (2017) study, the genes in our dataset were plotted. Figures show mean methylation level (β) in the corneal endothelium from non-FECD controls (dotted line) and FECD patients (continuous line). All genes are plotted in 5'→3' direction. Genomic positions are according to genome build GRCh37/hg19

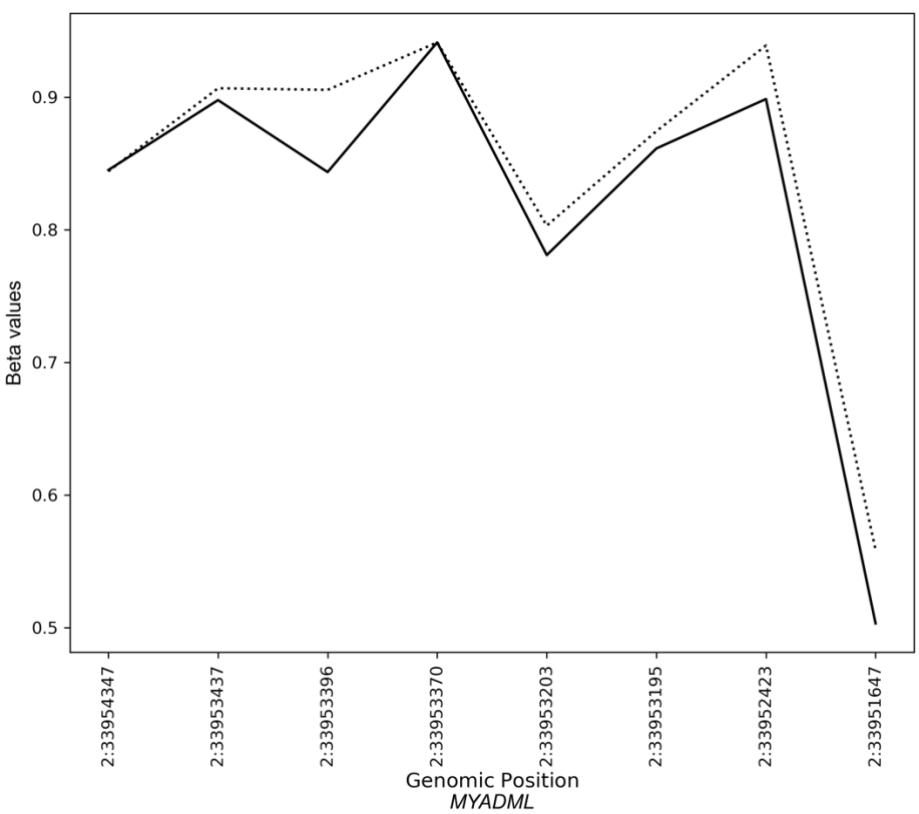

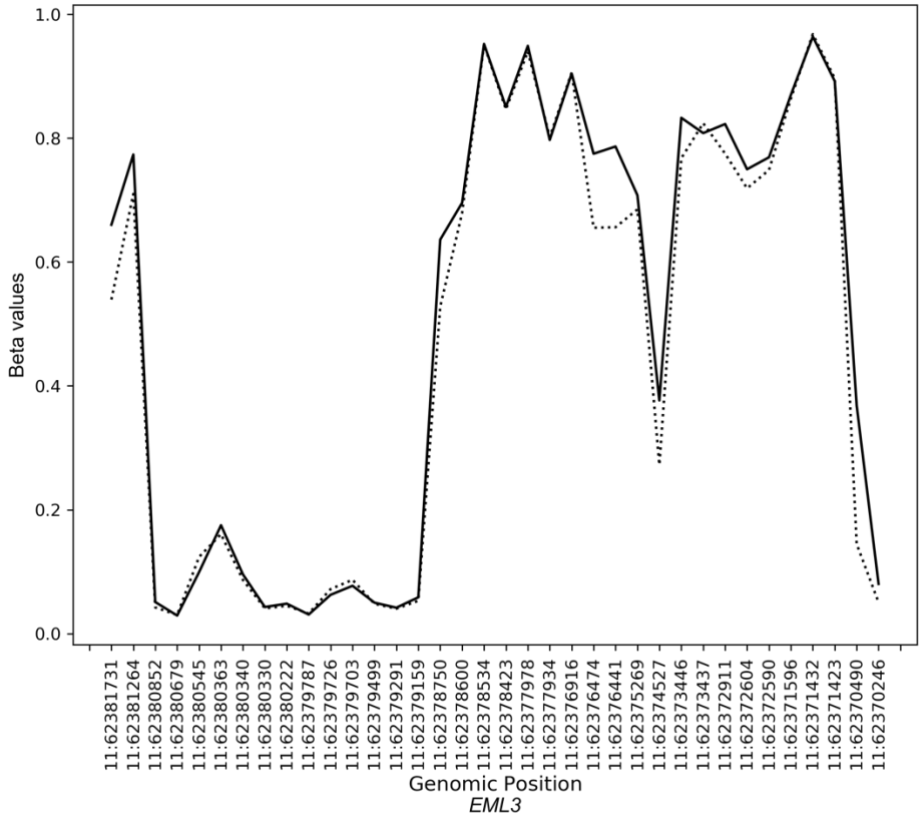

DNA methylation changes and increased mRNA expression of coagulation proteins, Factor V and Thrombomodulin in Fuchs endothelial corneal dystrophy in "Cellular and Molecular Life Sciences" by Westin IM, Landfors M, Giannopoulos A, Viberg A, Osterman P, Byström B, Degerman S, Golovleva I*.
*Correspondence to Irina Golovleva, Clinical Genetics, University Hospital, SE 901 85, Umeå, Sweden. E-mail_ irina.golovleva@umu.se

DNA methylation levels of genes containing CpGs overlapping with top 20 CpGs in Pan *et al*. (2019)

For probes in our dataset that overlap with top 20 probes from Pan et al (2019) study, the genes in our dataset were plotted. Figures show mean methylation level (β) in the corneal endothelium from non-FECD controls (dotted line) and FECD patients (continuous line). Vertical lines show standard deviation at each CpG site. All genes are plotted in 5'→3' direction. Genomic positions are according to genome build GRCh37/hg19

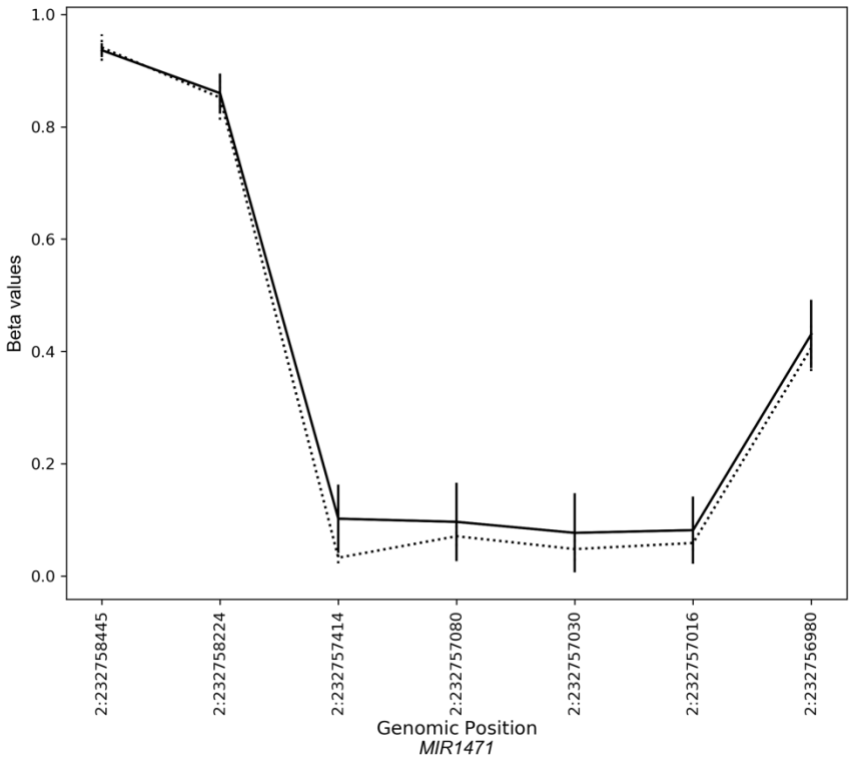

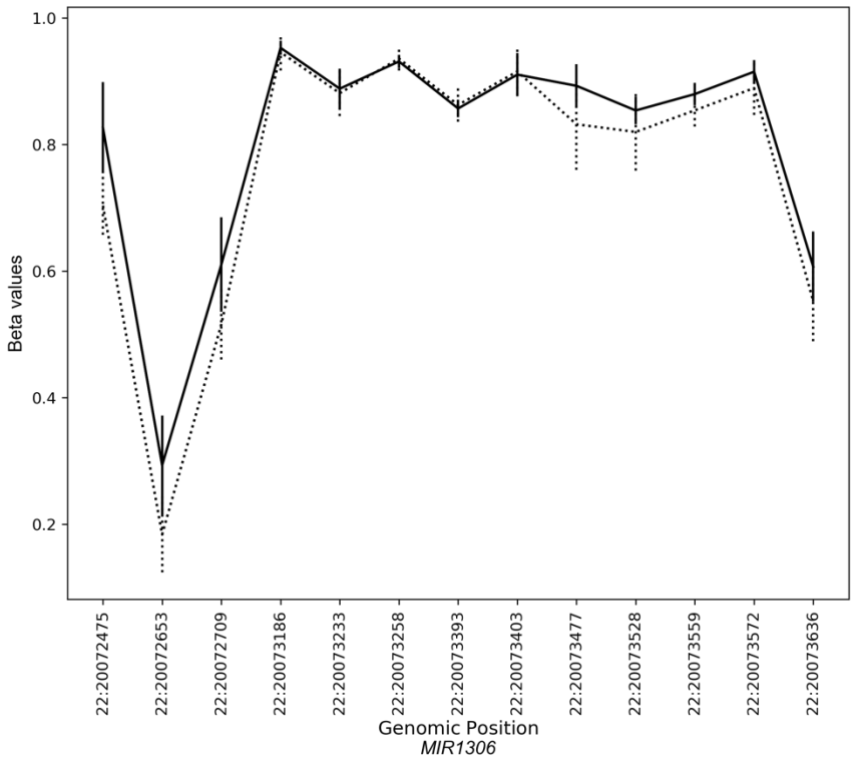

DNA methylation changes and increased mRNA expression of coagulation proteins, Factor V and Thrombomodulin in Fuchs endothelial corneal dystrophy in “Cellular and Molecular Life Sciences” by Westin IM, Landfors M, Giannopoulos A, Viberg A, Osterman P, Byström B, Degerman S, Golovleva I*.
*Correspondence to Irina Golovleva, Clinical Genetics, University Hospital, SE 901 85, Umeå, Sweden. E-mail_ irina.golovleva@umu.se

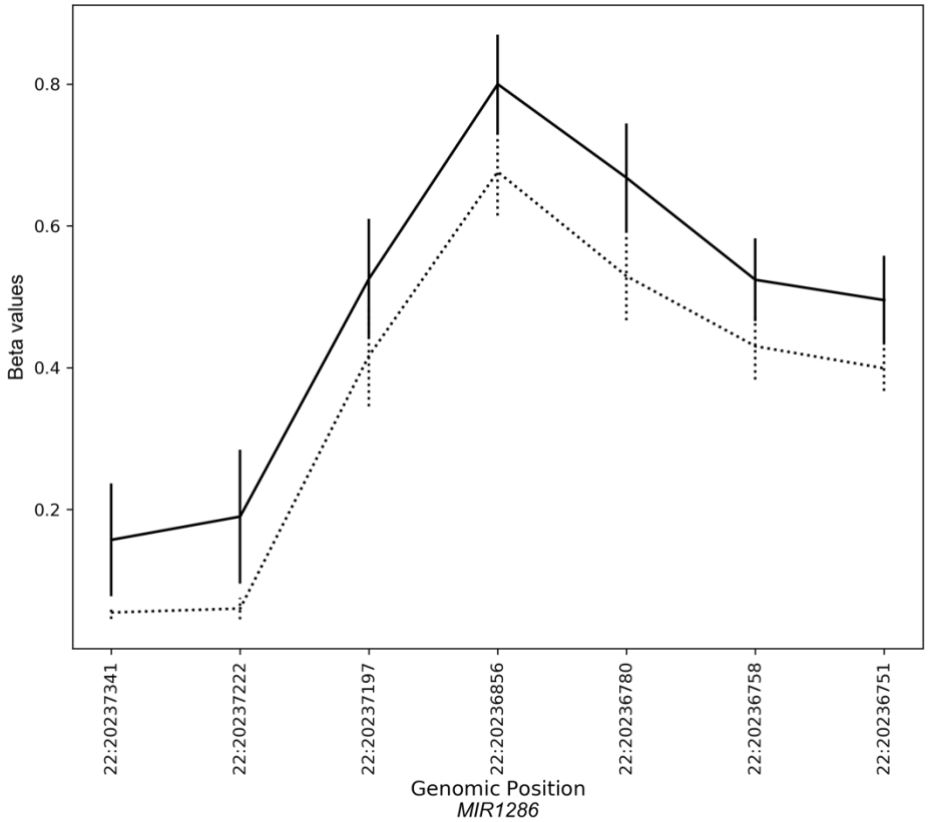

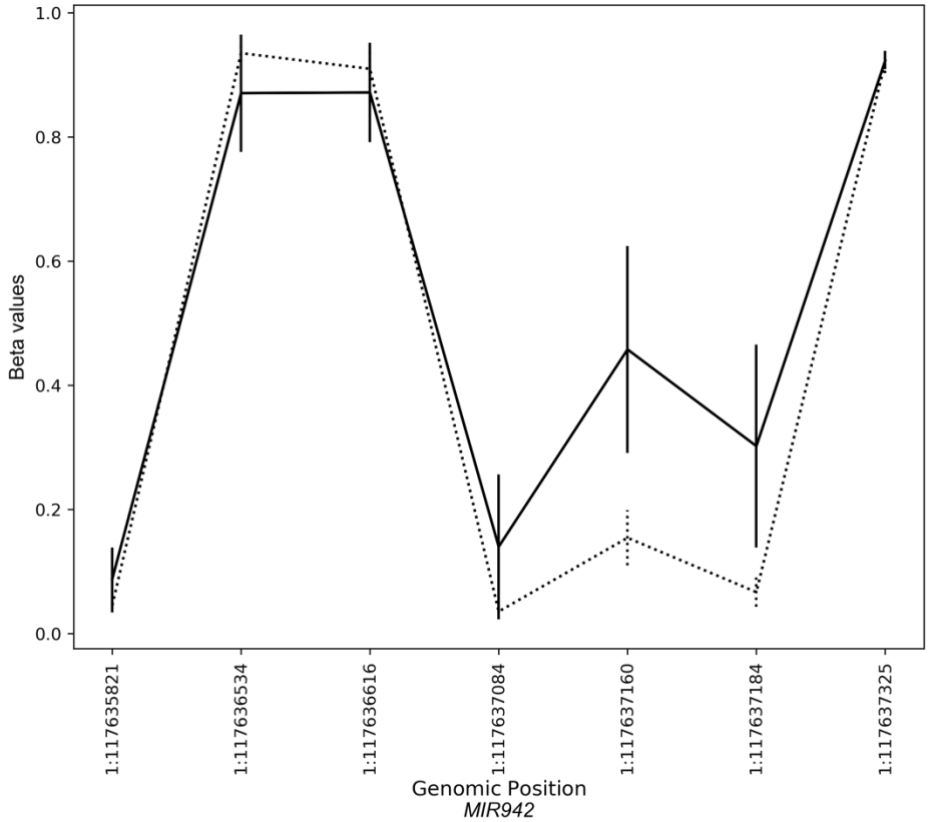

DNA methylation changes and increased mRNA expression of coagulation proteins, Factor V and Thrombomodulin in Fuchs endothelial corneal dystrophy in “Cellular and Molecular Life Sciences” by Westin IM, Landfors M, Giannopoulos A, Viberg A, Osterman P, Byström B, Degerman S, Golovleva I*.
*Correspondence to Irina Golovleva, Clinical Genetics, University Hospital, SE 901 85, Umeå, Sweden. E-mail_ irina.golovleva@umu.se

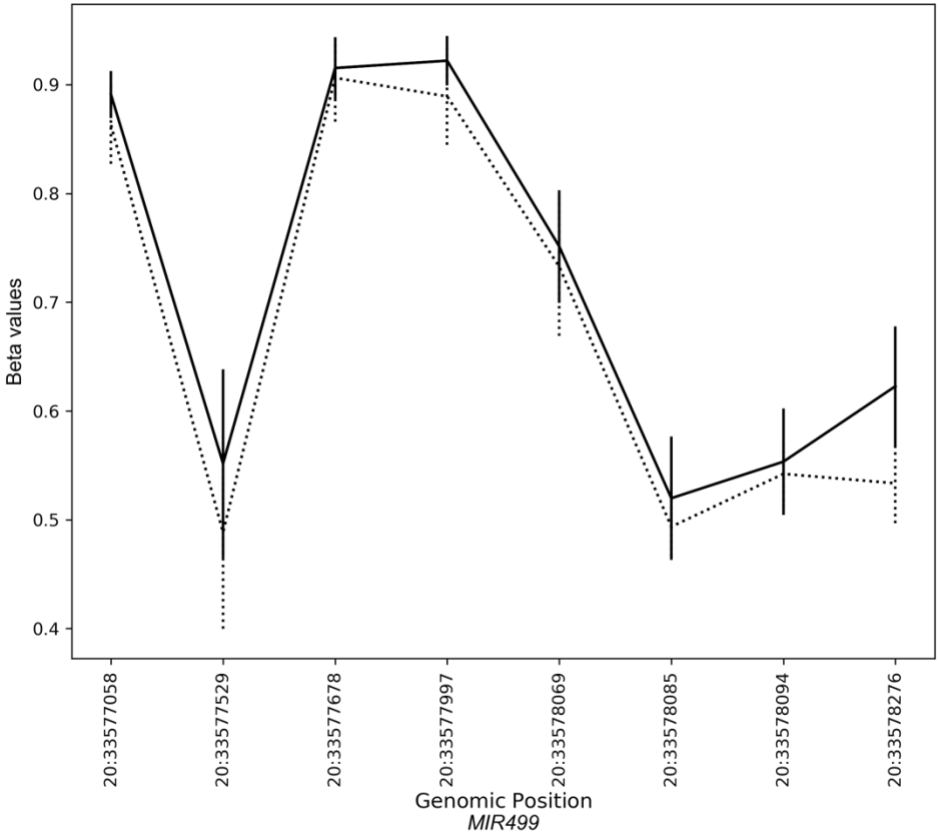

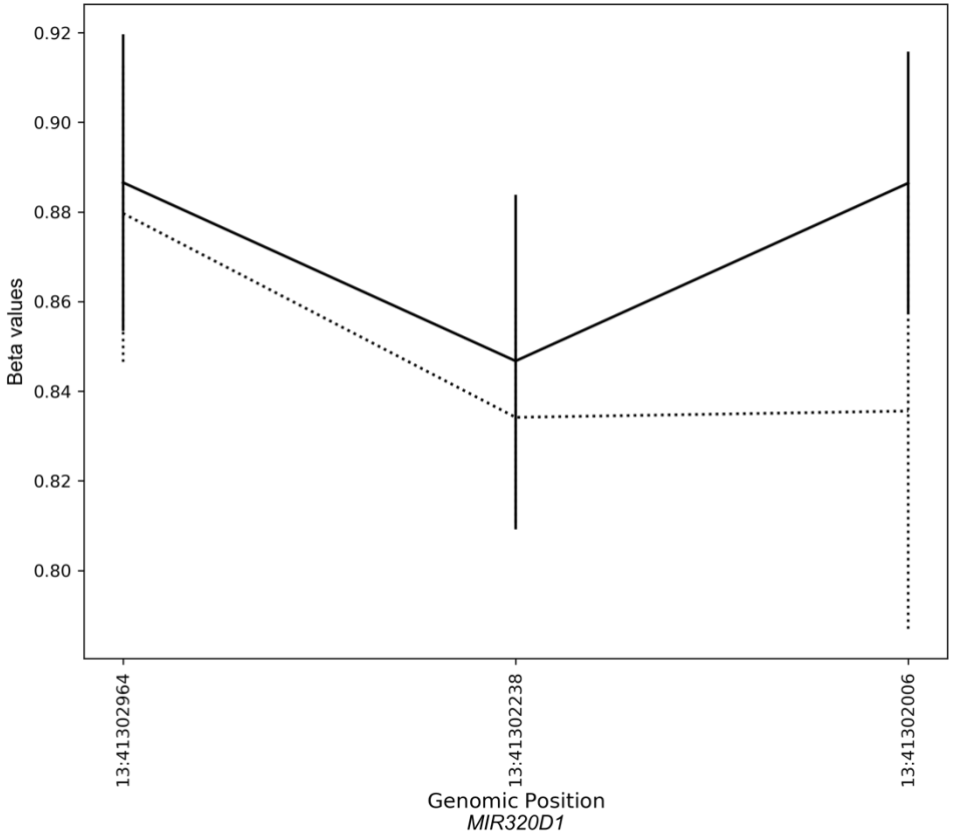

DNA methylation changes and increased mRNA expression of coagulation proteins, Factor V and Thrombomodulin in Fuchs endothelial corneal dystrophy in “Cellular and Molecular Life Sciences” by Westin IM, Landfors M, Giannopoulos A, Viberg A, Osterman P, Byström B, Degerman S, Golovleva I*.
*Correspondence to Irina Golovleva, Clinical Genetics, University Hospital, SE 901 85, Umeå, Sweden. E-mail_ irina.golovleva@umu.se

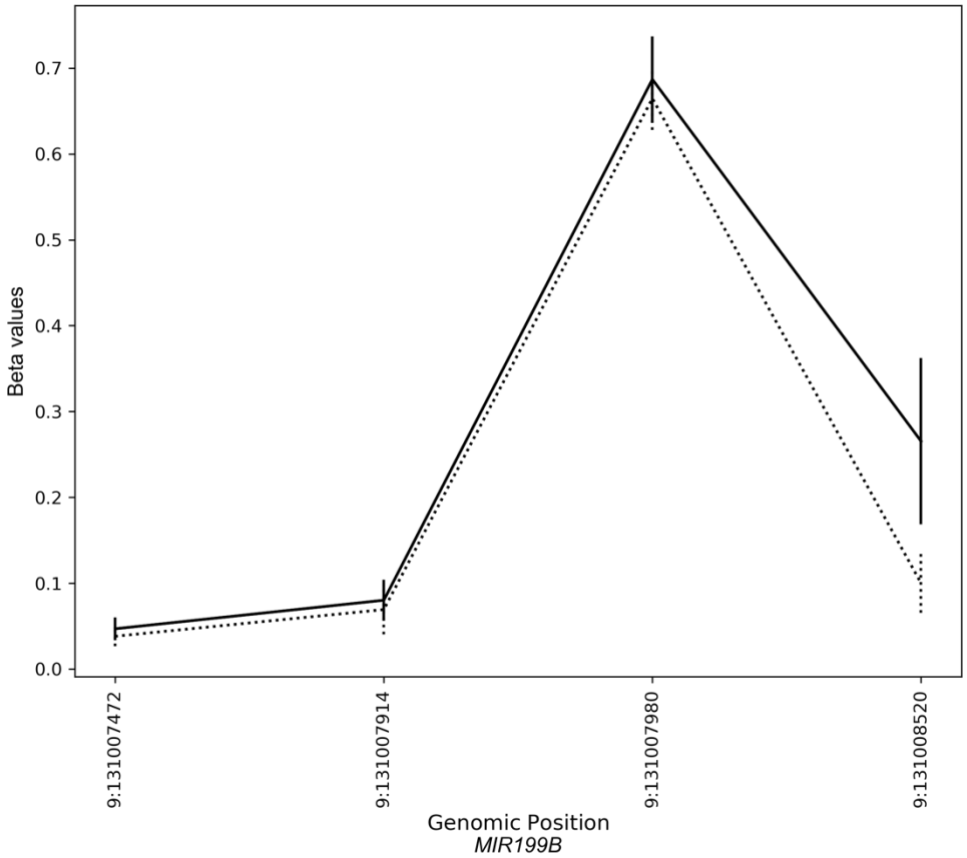

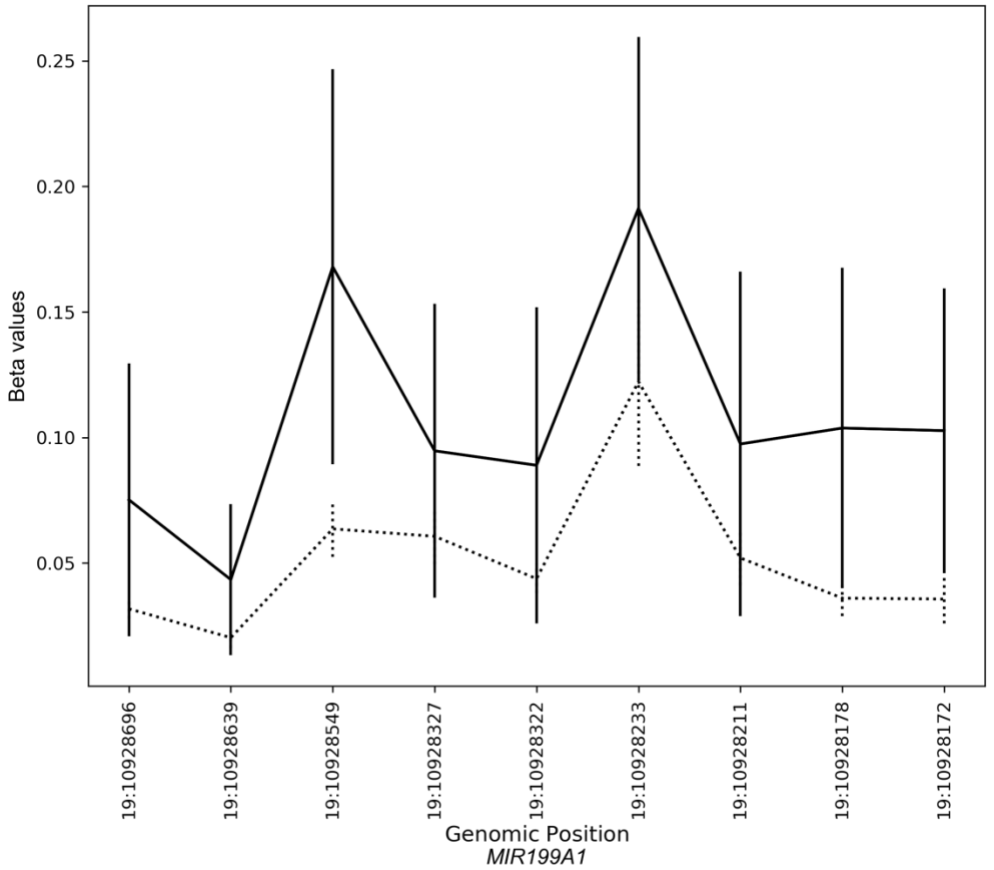

DNA methylation changes and increased mRNA expression of coagulation proteins, Factor V and Thrombomodulin in Fuchs endothelial corneal dystrophy in “Cellular and Molecular Life Sciences” by Westin IM, Landfors M, Giannopoulos A, Viberg A, Osterman P, Byström B, Degerman S, Golovleva I*.
*Correspondence to Irina Golovleva, Clinical Genetics, University Hospital, SE 901 85, Umeå, Sweden. E-mail_ irina.golovleva@umu.se

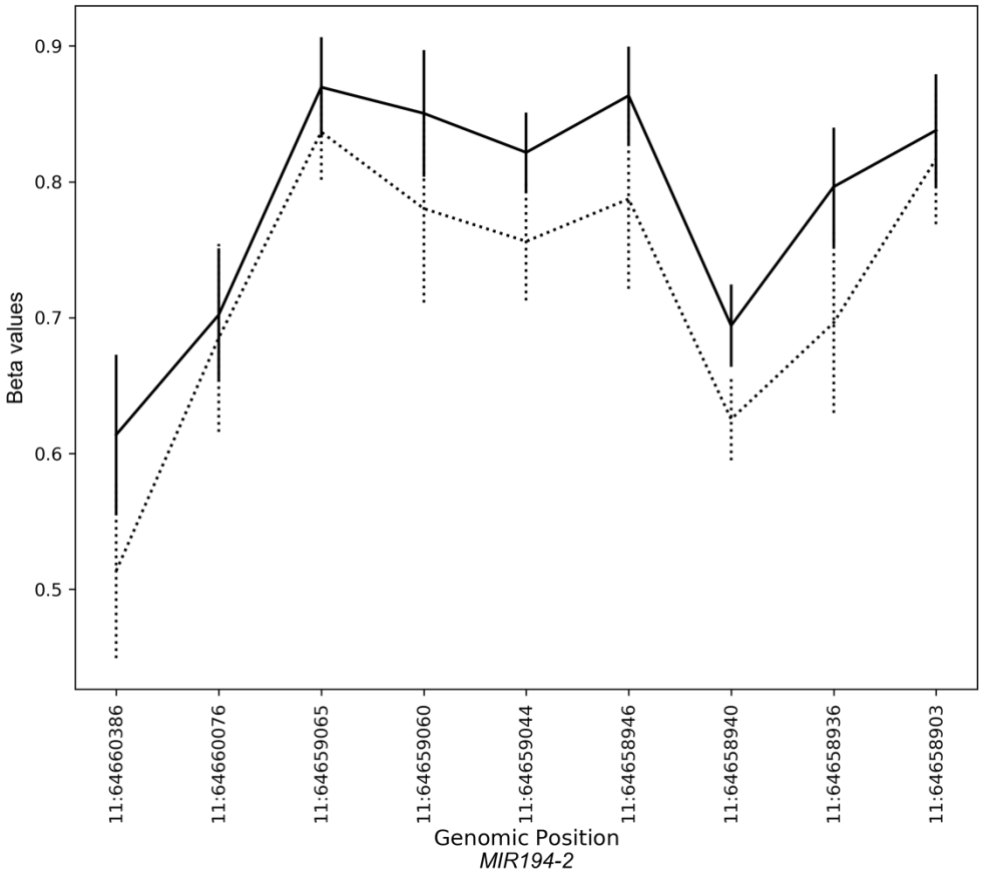

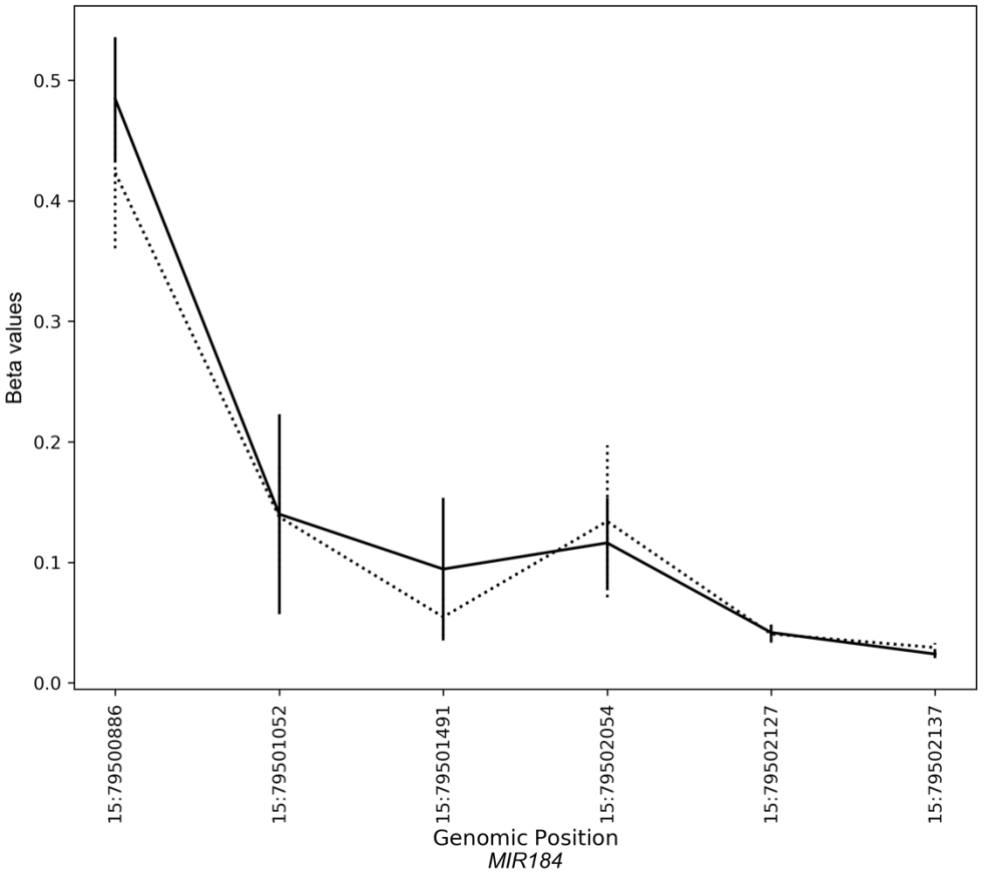

DNA methylation changes and increased mRNA expression of coagulation proteins, Factor V and Thrombomodulin in Fuchs endothelial corneal dystrophy in “Cellular and Molecular Life Sciences” by Westin IM, Landfors M, Giannopoulos A, Viberg A, Osterman P, Byström B, Degerman S, Golovleva I*.
*Correspondence to Irina Golovleva, Clinical Genetics, University Hospital, SE 901 85, Umeå, Sweden. E-mail_ irina.golovleva@umu.se

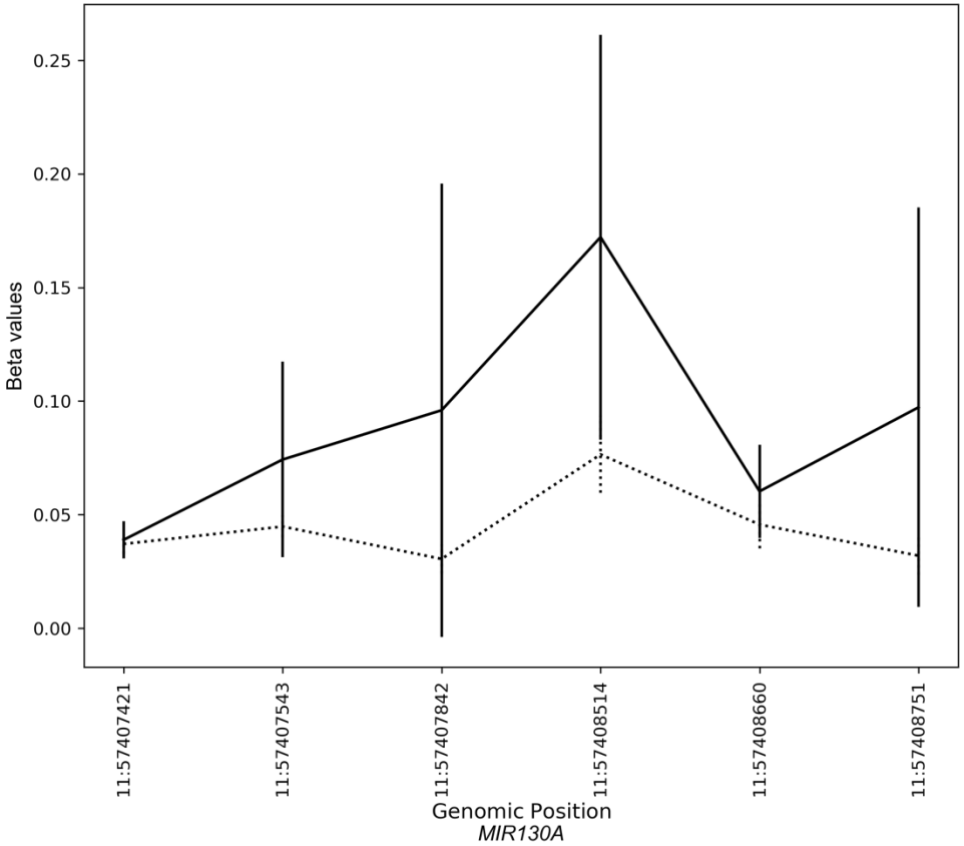

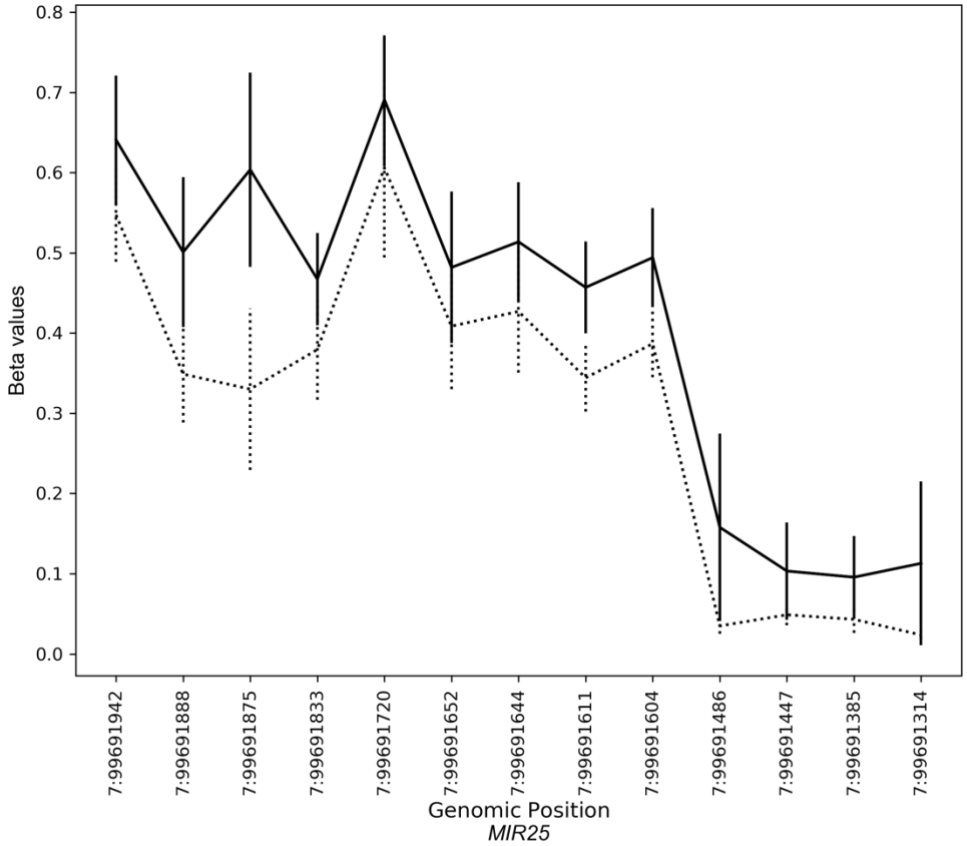

DNA methylation changes and increased mRNA expression of coagulation proteins, Factor V and Thrombomodulin in Fuchs endothelial corneal dystrophy in “Cellular and Molecular Life Sciences” by Westin IM, Landfors M, Giannopoulos A, Viberg A, Osterman P, Byström B, Degerman S, Golovleva I*.
*Correspondence to Irina Golovleva, Clinical Genetics, University Hospital, SE 901 85, Umeå, Sweden. E-mail_ irina.golovleva@umu.se

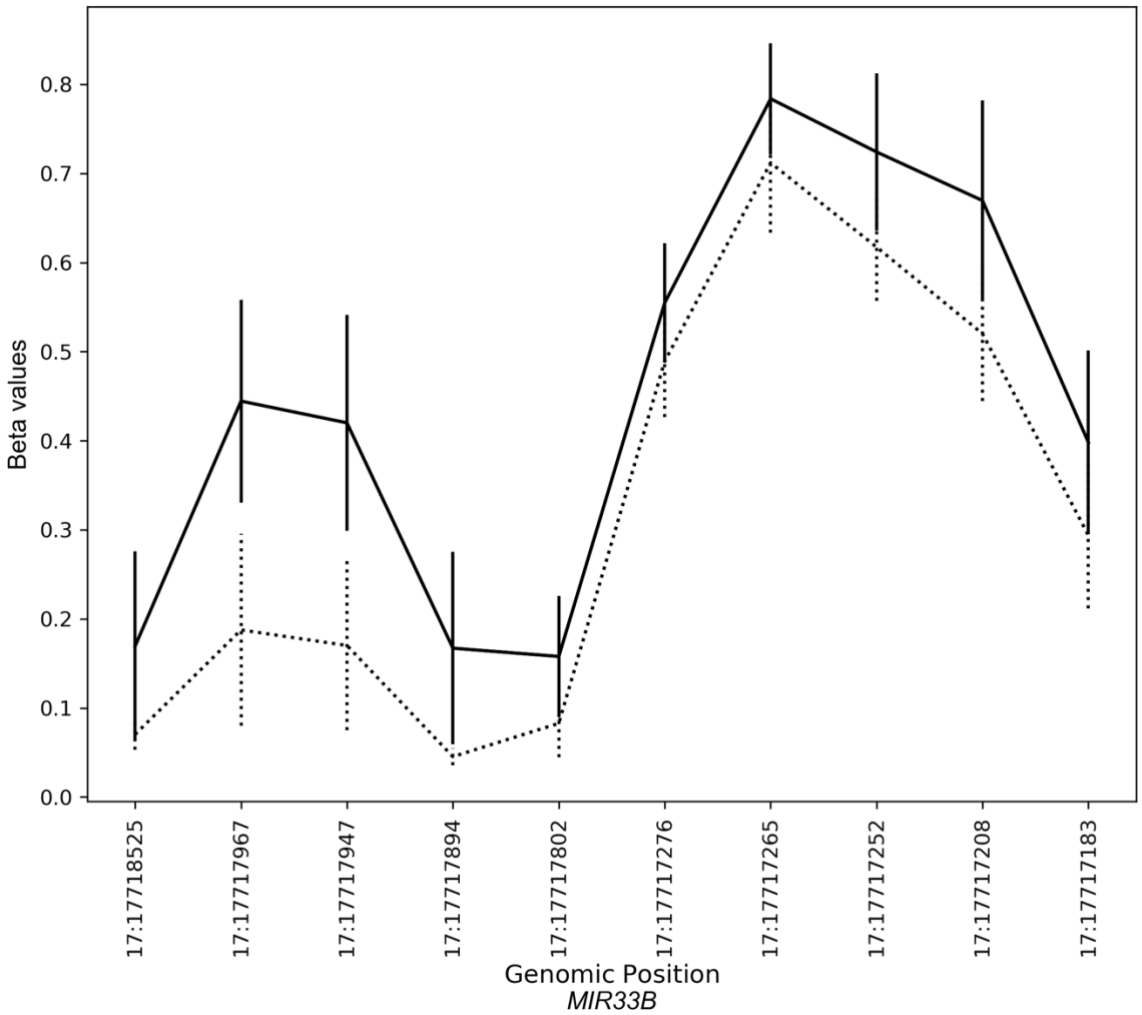

Supplement: Supplementary file 3 — Supplementary file3 (PDF 2852 KB) [file 18_2023_4714_MOESM3_ESM.pdf]
